# Engaging patients to improve quality of care: a systematic review

**DOI:** 10.1186/s13012-018-0784-z

**Published:** 2018-07-26

**Authors:** Yvonne Bombard, G. Ross Baker, Elaina Orlando, Carol Fancott, Pooja Bhatia, Selina Casalino, Kanecy Onate, Jean-Louis Denis, Marie-Pascale Pomey

**Affiliations:** 1 Institute of Health Policy, Management and Evaluation, University of Toronto, 155 College Street 4th Floor, Toronto, Ontario M5T 3M6 Canada; 2 Li Ka Shing Knowledge Institute, St. Michael’s Hospital, 30 Bond St, Toronto, Ontario M5B 1W8 Canada; 3 Niagara Health System, 1200 Fourth Avenue, St. Catharines, Ontario L2S 0A9 Canada; 4 Professor of Health Policy and Management, School of Public Health, Université de Montréal-CRCHUM & Canada Research Chair in Health System Design and Adaptation, 900, Saint Denis Street, Pavillion R, Montreal, Quebec H2X 0A9 Canada; 5 Départment de Gestion, d’Évaluation et de Politique de Santé, École de santé Publique, Université de Montréal, Centre de recherche du CHUM, Carrefour de l’innovation et de l’évaluation en santé, 850 rue Saint-Denis, Montréal, Quebec H2X 0A9 Canada

**Keywords:** Patient engagement, Patient involvement, Quality of care, Quality improvement, Health services, Health delivery, Systematic review

## Abstract

**Background:**

To identify the strategies and contextual factors that enable optimal engagement of patients in the design, delivery, and evaluation of health services.

**Methods:**

We searched MEDLINE, EMBASE, CINAHL, Cochrane, Scopus, PsychINFO, Social Science Abstracts, EBSCO, and ISI Web of Science from 1990 to 2016 for empirical studies addressing the active participation of patients, caregivers, or families in the design, delivery and evaluation of health services to improve quality of care. Thematic analysis was used to identify (1) strategies and contextual factors that enable optimal engagement of patients, (2) outcomes of patient engagement, and (3) patients’ experiences of being engaged.

**Results:**

Forty-eight studies were included. Strategies and contextual factors that enable patient engagement were thematically grouped and related to techniques to enhance design, recruitment, involvement and leadership action, and those aimed to creating a receptive context. Reported outcomes ranged from educational or tool development and informed policy or planning documents (discrete products) to enhanced care processes or service delivery and governance (care process or structural outcomes). The level of engagement appears to influence the outcomes of service redesign—discrete products largely derived from low-level engagement (consultative unidirectional feedback)—whereas care process or structural outcomes mainly derived from high-level engagement (co-design or partnership strategies). A minority of studies formally evaluated patients’ experiences of the engagement process (*n* = 12; 25%). While most experiences were positive—increased self-esteem, feeling empowered, or independent—some patients sought greater involvement and felt that their involvement was important but tokenistic, especially when their requests were denied or decisions had already been made.

**Conclusions:**

Patient engagement can inform patient and provider education and policies, as well as enhance service delivery and governance. Additional evidence is needed to understand patients’ experiences of the engagement process and whether these outcomes translate into improved quality of care.

**Registration:**

N/A (data extraction completed prior to registration on PROSPERO).

**Electronic supplementary material:**

The online version of this article (10.1186/s13012-018-0784-z) contains supplementary material, which is available to authorized users.

## Background

Patient engagement has become a cornerstone of quality of care [1–6] and is a frequently stated goal for healthcare organizations. Traditionally, and most commonly, this engagement has focused on the relationship between patients and providers in making care decisions or how to improve patient efforts to manage their own care [7]. However, there are growing efforts to integrate patients in broader ways, including efforts to improve or redesign service delivery by incorporating patient experiences [8–12]. These efforts are due in part to an increased recognition and acceptance that users of health services have a rightful role, the requisite expertise, and an important contribution in the design and delivery of services [4]. While the nature of patient engagement may vary from including patients as members of a board to time-limited consultation with patients on service redesign, its aims are consistent—to improve the quality of care [11, 13, 14].

Healthcare organizations have a long tradition of measuring the experience of patients, and health service “users” including families, caregivers, and clients, with their services. Yet, traditional satisfaction surveys often prove difficult to translate into improved service delivery [15, 16]. Indeed, research on patient engagement has pointed to the importance of augmenting traditional surveys and complaint processes, moving towards fuller engagement of patients in reviewing and improving the quality of service delivery in institutions and in the community [17–25]. This recognition has been accompanied by a growth in the development of instruments to measure and improve the quality of care patients receive. Over the past two decades, assessments of quality of care from the patient perspective have shifted from patient satisfaction to patient experiences [26]. Increasing literature indicates that it is not only feasible to involve patients in the delivery or re-design of health care [9] but that such engagement can lead to reduced hospital admissions [27], improved effectiveness, efficiency and quality of health services [28–31], improved quality of life, and enhanced quality and accountability of health services [9]. Frameworks of patient involvement have been developed that move from the traditional view of the patient as a passive recipient of a service to an integral member of teams re-designing health care [8, 11]. For example, one framework developed by Bate and Robert (2006) describes a continuum of patient involvement, which ranges from complaints, giving information, listening, and consulting towards experience-based co-design of services [8]. Low-level engagement, such as consulting, comprises largely unidirectional feedback (e.g., focus groups, surveys, interviews), whereas high-level engagement, like co-design, represents a partnership in the design or evaluation of services. A more recent framework developed by Carman et al. describes various levels of engaging patients and families in health and health care, from consultation or involvement to partnership and shared leadership in various activities including direct care, organizational design, and governance to policy-making [11]. Carman’s continuum of engagement was influenced by Arnstein’s formative “ladder of citizen participation,” a continuum of public participation in governance ranging from limited participation to a state of collaborative partnership in which citizens share leadership or control decisions [32].

**Fig. 1:**
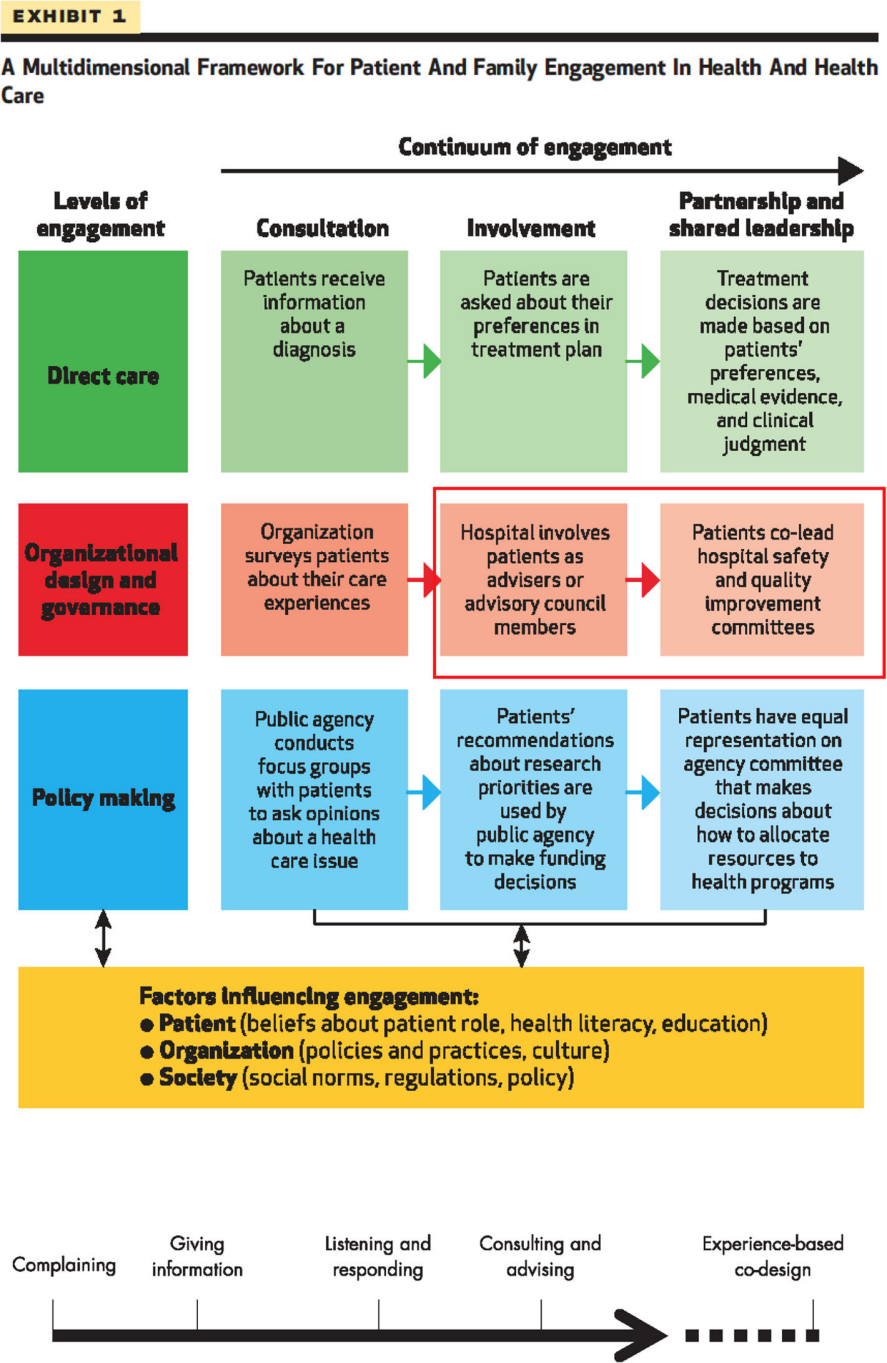
Patient engagement frameworks used for the selection and analyses of studies included in our review. The red box indicates the level of engagement along the continuum that is the focus of our studies included in our review [11]. The organizing framework used for analyzing the studies reviewed [8]

Governments and health care institutions are urged by some experts to engage patients and other service users, including caregivers and relatives in more robust ways [8, 33] where patients are actively involved as partners or co-leads in organizational re-design and evaluation of health care delivery, as depicted by the red section in Carman’s framework (Fig. 1). Despite the substantive body of research on strategies to engage patients and their effects on patients and health services, the literature is dispersed and has not been recently synthesized into a coherent overview. If the benefits of engaging patients in the design or delivery of health care are to be realized at an organization or system level, then effective strategies and the contextual factors enabling their outcomes need to be identified so that learning can be generalized. We conducted a systematic review of international English language literature on strategies for actively engaging patients and families in improving or redesigning health care and the contextual factors influencing the outcomes of these efforts. The explicit questions that guided our review were:

1. What are the strategies and contextual factors that enable optimal engagement of patients in the design, delivery, and evaluation of health services?
2. What are the outcomes of patient engagement on services?
3. What are patients’ experiences of being engaged?

## Methods

### Approach

**Fig. 2:**
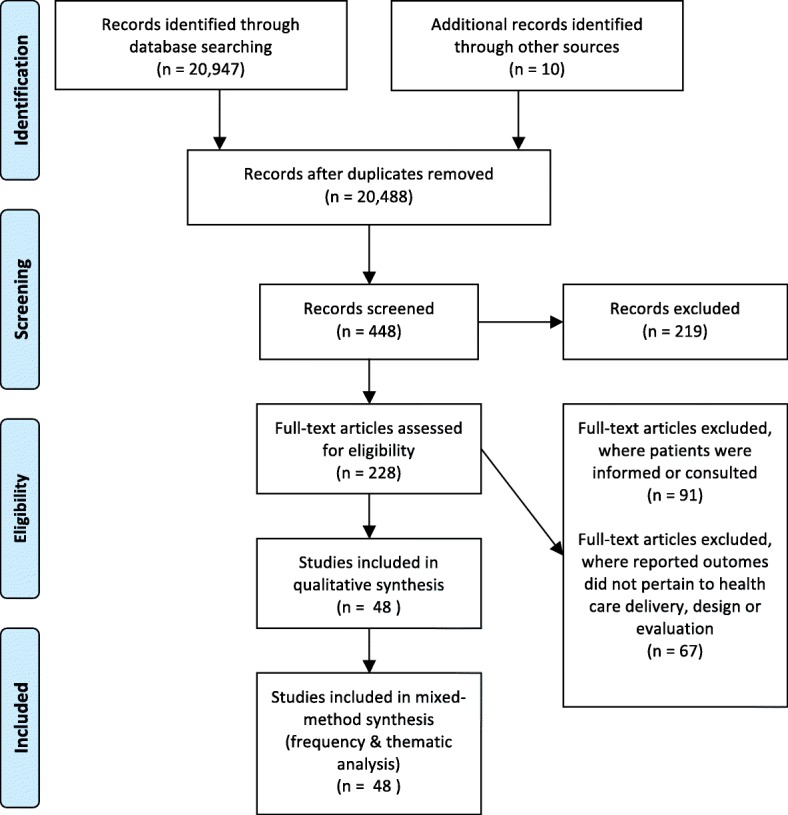
Flow diagram for search and selection process. From: Moher D, Liberati A, Tetzlaff J, Altman DG, The PRISMA Group (2009). Preferred Reporting Items for Systematic Reviews and Meta-Analyses: The PRISMA Statement. PLoS Med 6 (6): e1000097. 10.1371/journal.pmed1000097

We took a comprehensive approach in our systematic search and included all empirical qualitative, quantitative, and mixed methods study designs across all settings of care to address our narrow research questions. Our review did not fit into typologies of literature reviews [34, 35], given that we included qualitative and quantitative studies (to capture the breadth of studies in this area), employed a thematic analysis (given the multiplicity of designs), and applied a quality appraisal. We followed the PRISMA reporting criteria for Systematic Reviews and Meta-Analyses (Fig. 2) [36].

### Search strategy

In accordance with the core principles of systematic review methodology [37], we conducted a systematic review of relevant literature with the help of a librarian using the electronic databases of: MEDLINE, EMBASE, CINAHL, the Cochrane Library, Scopus, PsychINFO, Social Science Abstracts, AbiInform Business Source Premier (EBSCO), and ISI Web of Science. We searched the databases using the following subject headings related to patient engagement—combinations of “patient”, “user”, “client”, “caregiver”, “family” *and* “engage*”, “participat*”, “involve*”, “consult*”; for those related to designing, evaluating and delivery of services*—*combinations of “design”, “deliver*”, “evaluat*”, “outcome”, “develop*”, “plan*” *and* “health services”, “health care”, “health”, “service”. We included a combination of search terms from each category for each search, for example, “patient” AND “engage*” AND “design” AND “health services”).

### Criteria for selection

Studies were eligible for inclusion if they were available empirical articles that explicitly investigated the participation of patients, caregivers, or families in the design, delivery, and evaluation of health services, which aligns with involving or partnering/sharing leadership with patients in organizational design and governance, reflecting Carman’s framework (Fig. 1) [11]. Searches were restricted to qualitative, quantitative, or mixed methods articles published in English between January 1990 and March 2016. We chose 1990 as this coincided with the emergence of patient engagement particularly in mental health services and the broader quality of care discourse. All settings of care were eligible. We excluded articles that did not explicitly address patient engagement, as well as those that did not pertain to the broader design, delivery, and evaluation of health services (e.g., directly engaging patients in patient safety activities such as challenging staff who treat them to wash their hands or monitor the use of a safety checklist in their care, or in their self-management or treatment decisions, or studies pertaining to patient involvement in health research, community development, or health promotion). We also excluded articles that did not describe the outcome of the engagement of patients and those in which the outcomes did nor pertain to the design, delivery, or evaluation of health services (e.g., those that related to developing questionnaires or conceptual frameworks, insights on how to engage patients or work collaboratively). We focused on studies that consulted, involved, partnered, or co-designed health services with patients, informed by Bate and Robert’s [8] and Carman et al.’s [11] frameworks on patient engagement (Fig. 1). Finally, theoretical or conceptual articles as well as those focused on guideline development, instrument development, or broader organizational issues were excluded.

Titles and abstracts of the papers were examined to decide if the full article should be retrieved (Fig. 2). EO and CF were the primary reviewers who examined the titles and abstracts, applied inclusion criteria to the articles, and abstracted the data using an abstraction form. Any disagreement and uncertainties regarding inclusion were discussed and agreed upon by an additional reviewer (YB) on the abstraction form. We conducted calibration exercises to ensure reliability in applying the selection criteria. Reviewers independently screened the titles and abstracts, and discrepancies were discussed and reviewed by the third reviewer. There was a 95.46% observed agreement and 85.75% expected agreement between primary reviewers, with a kappa statistic of 0.703 (standard error, 0.021; 95% confidence interval, 0.662–0.744), which is relatively high compared to other knowledge synthesis protocols reporting 50% consistency rates [34].

### Data abstraction and synthesis

Data abstraction forms were used to describe the studies’ population, location (i.e., country), goals, methodology, and outcomes (Table 1); contextual factors influencing engagement (i.e., leadership and specific barriers and facilitators to patient engagement) (Table 2); and patients’ experience with the engagement and evaluation of study quality (Tables 2 and 3). Studies were then categorized by the level of patient engagement using Bate and Robert’s (2006) continuum of patient involvement [8]. Consistent with our aims to review strategies for actively engaging patients and families in improving or redesigning health care, we focused on studies using co-design or those consulting patients but also using elements of co-design—i.e., the more active levels of engagement on the Bates and Robert continuum. We classified changes or products of engaging patients as “quality of care outcomes” and the impact of the engagement on patients as “patients experience outcomes” (Table 1). Quality of care outcomes were categorized into one of the following: developing education or a service-related tool, informing policy or planning documents, and enhancing services or governance. Study quality was assessed by one person and two verifiers using a quality appraisal tool that systematically reviews disparate forms of evidence and methodologies on a scale from “very poor,” “poor,” “fair,” and “good” [38], which reflected the mixed methods articles in our review. Verification involved systematically checking and confirming the fit between each criterion of the assessment tool and the conceptual work of analysis and interpretation of study quality among a subset of studies. We also assessed the possible impact of study quality on the review’s findings (akin to a “sensitivity analysis” conducted for meta-analyses).

**Table 1:** Overview of patient engagement studies to improve quality of care

Study	Country	Intervention	Type of service	Sample size	Level of engagement	Outcomes on quality of care	Impact on institution
Acri et al. 2014 [65]	USA	Community collaboration model	Mental health services	16	Consultative to co-design	Developed and implemented screening, education, and an empowerment intervention for peer-delivered services targeted at improving emotional health of low-income new mothers	Users had an equal voice throughout all aspects of building the intervention, which equalized the power differential
		Meetings					
Barnes 2000 [75]	UK	Document analysis interviews questionnaire	Mental health services	Not clear	Co-design	Developed a new program	Achieved a culture change towards valuing users’ knowledge, expertise, contributions, and greater power-sharing
Barnes and Wistow 1994 [40]	UK	User panels	Community mental health and disabilities services	Not discussed	Consultative with some co-design	Identified strategies for user involvement; some evidence of service improvements; developed a “change agenda”	Achieved a culture change via “top-down” approach from local authority
Berg et al. 2015 [50]	Norway	User board	Community HIV/AIDS prevention	7	Co-design	Created a design for an outpatient clinic	Empowerment and autonomy of users through “active citizenship” and a “egalitarian spirit”
Blickem et al. 2013 [92]	UK	Focus groups	Mental health services	8 in interviews	Consultative to co-design	Developed and tested a protocol for PLANS, a community-based referral system	Close engagement of potential users resulted in a grassroots understanding of the support valued by individuals
		Interviews					
		Surveys		6 focus groups (total number not discussed)			
Bone et al. 2013 [93]	USA	Community-academic advisory committee	Cancer screening for African Americans	Not discussed	Co-design	Developed a community health worker model to engage African-American communities in cancer screening and care	Identified the community-academic advisory committee as vital to designing the service and ensuring its effectiveness
Brooks 2008 [64]	UK	Focus groups	General health services	52	Co-design	Patient involvement in auditing processes, development of patient questionnaires, policies, and frameworks	Reinforced the importance of patient narratives and knowledge in organization and delivery of health care
		Interviews					
		Observations					
Buck 2004 [76]	US	Citizen advisory board	General health services (for homeless individuals)	7	Consultative to co-design	Developed informational brochures and recommendations for local interventions and services to improve general health services for the homeless	Achieved collaboration and mutual education
Carlson and Rosenqvist 1990 [51]	Sweden	Consultation meetings	Diabetes care	243	Consultative to co-design	Implementation of care improvement programs and patient information	Identified problems and steps to solve them
		Interviews					
		Training course					
Cawston 2007 [69]	UK	Focus groups	Primary care	72 in focus groups; 372 via questionnaires	Consultative to co-design	Recommendations and some changes made to improve diabetes management in primary care	Created research-community partnerships in evaluating services but had a modest impact on service change
		Interviews					
		Questionnaire					
Coad 2008 [41]	UK	Youth Council	Pediatrics—acute care youth services	17	Co-design	Demonstrated impact of youth council on specific areas of improvement	Identified ways of promoting further involvement
Coker et al. 2014 [72]	USA	Community advisory board	Pediatrics	3	Co-design	Developed care models for well-child care	Not discussed
Elwell 2014 [48]	UK	Group meetings	Acute care	Not discussed	Consultative to co-design	Developed and implemented care pathways for cellulitis care in the hospital	User involvement created the desire to change in the organization
							Aligned user involvement with strategic directions
Ennis et al. 2014 [70]	UK	Focus groups, interviews, service user planning committee, surveys, usability testing	Mental health services	121 users via surveys, unclear number via focus groups, 8 users via usability testing, 4 users via service user planning committee	Consultative to- co-design	Developed electronic personal health record for mental health patients	Not discussed
Enriquez et al. 2010 [67]	USA	Focus groups questionnaires	HIV and intimate partner violence prevention	7 user partners in design; 31 participants in feasibility study	Co-design	Feasibility of new service was established, which improved protective health behaviors, self-esteem, social support, and attitudes towards partner violence	Delivery of intervention was deemed feasible, community-provider partnership was well received and enhanced acceptability of the intervention
Erwin et al. 2016 [71]	USA	Focus groups	Pediatrics—asthma	20	Consultative to co-design	Developed new protocol and tool for patient discharge	Collaborative model enhanced the perception of ED clinicians as partners in asthma control
		Interviews					
		Surveys					
Factor 2002 [57]	USA	Focus groups	Substance users	29	Co-design	Development of a “survival guide” to improve access to treatment	Created and maintained the participation of users in all aspects of guide development
Ferreira-Pinto 1995 [58]	Mexico	Interviews	Community HIV/AIDS prevention	105	Co-design	Development and implementation of prevention program	Increased self-efficacy and self-esteem of community partners, beyond the program’s outcomes
		Questionnaires					
Fitzgerald 2011 [78]	UK	“Serious game”	Mental health services	25	Consultative to co-design	User re-design of layout and furnishings of a new service unit; design of a medication dispensing system	Achieved flexibility and inclusivity through a game format
Frazier 2007 [42]	USA	School-based program	Mental health services	Not discussed	Co-design	Developed a school-based mental health service program with active engagement of community partners and clinicians	Achieved successful collaboration between community and clinicians on curriculum development
Gibson 2005 [60]	UK	Interviews, questionnaires, workshops	Pediatric oncology	40	Consultative to co-design	Recommendations for structure and processes of service	Not discussed
Godfrey et al. 2013 [73]	UK	Facilitated workshops, focus groups, interviews	Acute care—delirium	3 delirium prevention team members, unspecified interviews	Consultative to co-design	Prevention of Delirium (POD) program	Enhanced culture of caring among staff
Hall 2011 [94]	UK	Interviews, modeling exercise	Cancer	18	Consultative to co-design	Developed a model for shared care of secondary cancer follow-up with general practitioners supported by specialists	Involved service users and deliverers’ experiences in a modeling exercise
Higgins et al. 2016 [95]	Ireland	Action research group, focus groups, peer facilitator, steering committee	Mental health services	30 users via focus groups, 21 family members via focus groups	Consultative to co-design	Developed a peer and clinician-led education program	Not discussed
Hopkins and Neimiec 2006 [52]	UK	Interviews questionnaires	Home treatment services	70	Co-design	Service improvement survey	Shared and neutralized power to increase inclusiveness through user participation in process
Iedema et al. 2010 [39]	Australia	Interviews	Emergency health services	40	Co-design	Recommendations for improving processes and facilities in the ED	Created a deliberative space for patients, clinicians, and staff to discuss personal experiences and design processes to ameliorate issues. Process developed new competencies and skills among participants
Jones et al. 2008 [62]	UK	Focus groups, interviews, workgroups	Stroke services	92	Consultative to co-design	Information package for patients, recommendations for improvements to rehabilitation program; prioritization of health care issues for stroke patients and development of services	Achieved meaningful user participation in service development through external facilitation
Jones 2010 [96]	USA	Advisory meetings interviews,user testing	Smoking cessation for deaf individuals	10	Consultative to co-design	Developed and tested an Internet-based smoking cessation intervention for deaf individuals in consultation with members of the deaf community	Involved service users in all phases of development and testing
Lofters et al. 2015 [43]	Canada	Community advisory group, community workshops, concept mapping, interviews	Cancer screening	24 via concept mapping	Consultative to co-design	Developed a culturally appropriate cancer screening program for South Asian community delivered via community organizations	Increased capacity to implement evidence-based interventions
Lord et al. 1994 [63]	Canada	Document analysis, consultation feetings, focus groups, questionnaire	Mental health services	Not discussed	Co-design	Improved services	Achieved organizational culture change and patient representation on board by broadening power and control through stakeholder involvement
Macdonnell et al. 2013 [45]	Canada	Brainstorming, facilitated discussions	Pediatrics—neonatal intensive care	3	Co-design	Developed a family integrated care program	Built positive relationships between users and staff
MacNeill 2009 [97]	UK	Interviews, observations	Pediatrics	29	Consultative to co-design	New model of participation to improve patient-staff relationship and patient understanding of program	Greater involvement of users through democratic process of participation, though users adopted a passive role
Mendenhall et al. 2010 [77]	USA	Collaborative educational program	Diabetes	52	Consultative to Co-design	Collaborative design of a “Family Education Diabetes Series” program, which demonstrated improved outcomes	Achieved collaboration between elders and providers in design and implementation of program through use of talking circles, storytelling, dance, shared meals, and active role in intervention
Murphy et al. 2015 [44]	Ireland	Quality improvement working teams	Mental health services	10	Co-design	Enhanced experiences of care for users referred to community mental health services	Acknowledgement that user/family involvement needs to go beyond involvement to true co-production exercises perceived as meaningful by all participating stakeholders
Owens 2011 [56]	UK	Workshops	Mental health services	12	Co-design	Developed a text-based intervention for patients who self-harm	Involved users in the design process, which changed the nature of the intervention dramatically
Pilgrim and Waldron 1998 [59]	UK	Consultation meetings, observations	Mental health services	14	Co-design	Improved service: extended opening hours, employed a mental health advocate, published an information booklet	Achieved direct negotiations for change between users and professionals
Reeve et al. 2015 [74]	Australia	Focus groups, workshops	Primary care	6	Co-design	Generated new delineation of roles and responsibilities between an Aboriginal community-controlled health service and local Australian health service	Trusting relationship between community and providers as a result of extensive community consultation
Rose 2003 [98]	UK	Questionnaire	Mental health services	221	Consultative to co-design	Improved coordination of care generally linked to improved user satisfaction	Very few users were involved or aware of the new coordination process
Swarbrick et al. 2006 [55]	USA	Group meetings	Mental health services	Not discussed	Co-design	Implementation of the Recovery Network Program, a user-led wellness and recovery training project	Established a collaborative partnership between peer education and hospital staff via user training
Thomson et al. 2015 [68]	UK	“Future” groups	Multiple sclerosis	5	Co-design	Reconceptualized service for outpatients	Created a positive working environment with mutual respect and in equal partnership
Todd et al. 2000 [47]	UK	Interviews	Intellectual disability services	Not discussed	Co-design	Influenced implementation of service strategy	Achieved a shift in thinking, collaboration and consumer participation in planning
Tollyfield 2014 [53]	UK	Co-design meetings	Acute care—critical care	19	Co-design	Multiple in-unit quality improvement initiatives	Staff reconnected core values of caring and compassion
Tooke 2013 [49]	UK	Service user review panels	Dementia	14	Consultative to co-design	Development of organizational priorities and processes for patients with dementia, development of evaluation tools	Enhanced understanding of effective ways for staff to communicate with users
Van Staa et al. 2010 [66]	Netherlands	Interviews at a disco party	Acute care for chronically ill patients	34	Co-design to consultative	Recommendations for engaging youth in design and evaluation of health services	Involving users was feasible and appreciated by users but did not improve quality
Walsh and Hostick 2005 [99]	UK	Questionnaire	Mental health services	10	Consultative to co-design	Improved care facility, development of service strategy, and care guide	Achieved user ownership through external facilitation
Weinstein 2006 [46]	UK	Document analysis, meetings, questionnaire	Mental health services	72	Consultative to co-design	Plan to improve service delivery	Top-down approach of the first case resulted in less user ownership, whereas the collaborative, user-led approach of the second case led to the new approach to seeking users’ views and achieved higher response
Wistow and Barnes 1993 [61]	UK	Consultation meetings, patient council, questionnaire	Mental health and disability services	Not discussed	Co-design	Improved access to services: commitment to address issue, employment support unit created	Increased users’ voice in their care, which improved the sensitivity of services to individual needs and information about services
Xie et al. 2015 [54]	USA	Interviews, meetings	Acute care	1 parent, 14 stakeholders	Co-design	Developed checklist for family-centered rounds	Created buy-in for the family-centered rounds process and need for mutual understanding

**Table 2:** Summary of facilitators and barriers of patient engagement

Facilitators	Barriers
Design of engagement	
1. Techniques for enhancing patient/carer input	
● Enable patients or carers to set the agenda ● Enable patients or carers to participate in all/most stages of the research (participatory action research) ● Include higher proportions of patients versus providers to enhance patient voices ● Offer flexibility in the levels and approaches of involvement ● Build in reward mechanisms such as feedback and evaluation ● Set opportunities for interaction at regular frequencies	● Overly complex discussions ● Onerous, time-intensive involvement ● Inclusion of: ○ A disproportionate number of patients compared to providers ○ Providers who previously cared for the patients in the meeting/committee ○ Groups of individuals with existing hierarchical structures
2. Creating a receptive context	
● Use of democratic dialog to build consensus ● Use of external facilitation and trained facilitators ● Conduct training sessions prior to engagements to clarify roles, objectives, develop skills, increase sensitivity to cultural or community issues and reduce power imbalances ● Maintain flexibility in aims, design, and outcomes in response to patients’ input ● Enable time to develop strong and trusting relationships ● Create environment where participants are able to communicate in the language of their preference	● Lack of clarity on: ○ Roles ○ Objectives ○ Responsibilities
3. Leadership actions	
● Secure institutional commitment and sponsorship for engagement ● Involve institutional leadership ● Conduct engagements before decision have been made ● Establish mechanisms to act on issues raised and to continue involvement ● Demonstrate progress occurring between meetings	● Engagements conducted by consultative groups, not decision-makers ● Lack of response or plans to address issues raised ● Lack of follow-up with patients after their participation ● Policies and procedures misaligned with participation, recommendations or outcomes
Sampling of participants	
1. Techniques for enhancing patient/carer input	
● Have patients conduct interviews with fellow patients, when possible ● Strive for a wide representation of patients at all stages ● Identify and recruit users through providers, existing users, networks ● Offer incentives (monetary and other), stipends, reimbursement of expenses	● Provider- or patient-led recruitment can introduce biases ● Inclusion of self-selected, participants: ○ Confident patients ○ Those who have fewer symptoms or family care duties ● Inclusion of proxy groups: ○ Parents to represent children ○ Carers to represent patients ● Ethical concerns regarding recruitment and consent of participants with intellectual or physical disabilities
2. Creating a receptive context	
● Consider setting: engage patients at home, in their facilities or in environments outside where services are delivered to increase participation and comfort	● Lack of participant commitment ● Lack of participant confidence ● Inclusion of providers: ○ Who are skeptical towards involving patients ○ Who feel threatened by devolving power ○ Whose behavior does not promote user participation
3. Leadership actions	
● Emphasize to patients that there is organizational commitment/sponsorship of the engagement of patients

**Table 3:** Quality of care outcomes and levels of engagement

Type of outcomes	Level of engagement	Studies	
		Co-design	Consultative to co-design
Education or tool development			
Information packages for patients, peers, and providers	Consultative to co-design, co-design	Pilgrim and Waldron 1998 [59], Factor 2002 [57]	Jones 2008 [62], Carlson and Rosenqvist 1990 [51], Buck 2004 [76], Ennis 2014 [70], Erwin 2016 [71]
Service improvement surveys	Co-design, consultative to co-design	Hopkins and Neimiec 2006 [52], Brooks 2008 [64], Xie 2015 [54]	Tooke 2013 [49]
Informed policy or planning products			
Clinical care models	Consultative to co-design		Hall 2011 [94]
Service/care strategies	Co-design	Brooks 2008 [64]	
User involvement models	Co-design, consultative to co-design	Coad 2008 [41]	MacNeill 2009 [97], Van Staa 2010 [66], Barnes and Wistow 1994 [40]
Service policy implementation	Co-design	Todd 2000 [47]	
Plans or recommendations to improve service delivery and care	Co-design, consultative to co-design	Iedema 2010 [39]	Jones 2008 [62], Weinstein 2006 [46], Walsh and Hostick 2005 [99], Gibson 2005 [60], Buck 2004 [76], Cawston 2007 [69]
Enhanced care process or service delivery			
Extended opening hours	Co-design	Pilgrim and Waldron 1998 [59]	
Employment of a dedicated mental health advocate	Co-design	Pilgrim and Waldron 1998 [59]	
Improved/developed care facilities, services, programs, or intervention	Co-design, consultative to co-design	Ferreira-Pinto 1995 [58], Frazier 2007 [42], Barnes 2000 [75], Owens 2011 [56], Sawbrick 2006 [55], Lord 1998 [63], Coad 2008 [41], Fitzgerald 2011 [78], Macdonnell 2013 [45], Bone 2013 [93], Berg 2015 [50], Thomson 2015 [68], Reeve 2015 [74], Tolleyfield 2014 [53], Murphy 2015 [44]	Fitzgerald 2011 [78], Jones, 2010 [96], Mendenhall 2010 [77], Walsh and Hostick 2005 [99], Carlson and Rosenqvist 1990 [51], Barnes and Wistow 1994 [40], Cawston 2007 [69], Rose 2003 [98], Godfrey 2013 [73], Blickem 2014 [92], Acri 2014 [65], Higgins 2016 [95], Lofters 2015 [43]
Improved access to service	Co-design	Wistow and Barnes 1993 [61]	
Creation of an employment support unit	Co-design	Wistow and Barnes 1993 [61]	
Creation of new services	Co-design	Enriquez et al. 2010 [67]	
Improved governance			
Patient representation on board	Co-design	Lord et al. 1998 [63]	
Auditing policy and frameworks	Co-design	Brooks 2008 [64]	
Commitment to improve services	Co-design	Wistow and Barnes 1993 [61]	
Organizational culture change	Co-design	Lord et al. 1998 [63]	

### Data analysis

Data were analyzed to address the three research questions, with the intention of (1) identifying strategies and contextual factors that enable optimal engagement of patients in the design, delivery, and evaluation of health services; (2) identifying the outcomes of patient engagement; and (3) exploring patients’ experiences of being engaged. YB analyzed the data using quantitative (i.e., frequency analysis) and qualitative methods. YB used thematic analysis to identify the strategies and contextual factors (i.e., barriers and facilitators), outcomes, and experiences of optimal patient engagement. This process involved identifying prominent or recurring themes in the literature (relevant to our research questions) and summarizing the findings of different studies under thematic headings using summary tables. A coding framework was developed to thematically describe the strategies and contextual factors enabling patient engagement. YB and RB refined the framework as new data emerged during the analysis.

## Results

### Included studies

We found a total of 20,957 studies about involving patients in the design, delivery, or evaluation of health care. Of these, we excluded 20,909 because they did not report outcomes related to health care delivery, design, or evaluation (*n* = 67) or only informed/consulted with patients, as opposed to engage them in co-design (*n* = 91) (Fig. 2; Additional file 1: Table S3 & Additional file 2: Figure S1). Our final sample of studies included 48 papers involving patients, families, and caregivers along with service users, health care providers, staff, board members, health care managers, administrators, and decision-makers (Table 1). The publication date of the included studies spanned from 1993 to 2016, and interestingly, co-design was employed as early as 1993 to as recently as 2015 in published studies. Of the 48 included studies, 27 were qualitative studies; 3 were quantitative; 13 constituted mixed methods studies, which included qualitative, quantitative methods; and 5 comprised user panels or advisory meetings (Table 4). We restricted our analysis to articles actively engaging patients. Half of the articles (*n* = 24) included consultative activities typical of low-level engagement (i.e., where patients provided input on research design or measures as part of the research or administrative team). The other half were co-design (high-level engagement—i.e., deliberative, reflexive processes where patients and providers work together to create solutions [39]) (Table 4). Engagement efforts spanned a range of services, including pediatrics, community and primary care, and most frequently occurred in mental health services (*n* = 17; 35%—Tables 4 and 1). Studies originated from various countries, with most deriving from the UK (*n* = 26; 54%) (Tables 4 and 1). Few studies formally evaluated patients’ experiences of the process of being engaged (*n* = 12; 25%) (Additional file 3: Table S1).

**Table 4:** Characteristics of patient engagement studies

Study characteristics (*n* = 48)	Number	Percent
Country		
UK	26	54
USA	11	23
Canada	3	6
Australia	2	4
Ireland	2	4
Mexico	1	2
Sweden	1	2
Netherlands	1	2
Norway	1	2
Type of service		
Mental health	17	35
General health/community/primary care	5	10
Pediatric/maternity care	6	13
Acute care/emergency	6	13
Cancer	3	6
HIV/AIDS	3	6
Diabetes	2	4
Smoking cessation/substance abuse	2	4
Physical and intellectual disability	1	2
Elderly/home treatment	1	2
Stroke	1	2
Multiple sclerosis	1	2
Design		
Qualitative	27	56
Mixed methods	13	27
Quantitative	3	6
Other	5	11
Level of engagement		
Co-design	24	50
Consultative to co-design	24	50
Type of quality of care outcome*		
Discrete product		
Education/tool development	11	23
Enhanced policy or planning document	15	31
Care process or structural outcome		
Enhanced care process or service delivery	35	73
Enhanced governance	5	10
Evaluation of patient experiences of engagement process		
Formal	12	25
Informal; anecdotal reports	11	23
None	25	52
Evaluation of engagement methods		
Yes	25	52
No	23	48

*The total outcomes exceed the number of studies because some studies reported more than one outcome

### Strategies for optimal patient engagement to improve quality of care

We identified various strategies that contributed to optimal patient engagement, which were mediated by key contextual factors that enabled or constrained the effectiveness of the engagement. These strategies were thematically grouped as techniques to enhance (1) design, (2) recruitment, (3) involvement, (4) creating a receptive context, and (5) leadership actions. Here, we describe the strategies and contextual factors that enabled optimal patient engagement (see also Additional file 3: Table S1).

#### Techniques to enhance design of engagement

In designing engagements, several studies pointed to the importance of clarifying the objectives, roles, and expectations of the engagement for patients/carers [40–45]. Approaches that gave users specific roles or engaged them in a formal structure such as a steering committee [45] or that enabled patients to set the agenda, develop shared mission and purpose statements and participate in all/most stages of the planning, administration, and evaluation made participants feel comfortable with the team and process, maintained patient involvement throughout the course of the process, and improved the quality of outcomes [41, 45–50]. These techniques occurred in mental health, HIV, and pediatric service settings where patients were engaged to improve access to, and quality of, care or promote a culture change in the development and delivery of services.

An important strategy used in pediatric, diabetes, and home care settings was holding training sessions to prepare staff and patients, which provided clarity on roles and responsibilities, helped patients or carers understand how they could best contribute, sensitized participants to the contextual and cultural issues, and increased patients’ confidence and commitment to the engagement process [41, 51, 52]. Training also offered the benefit of building positive relationships between users, facilitators, and staff [43, 45, 49, 53, 54], which also served to mediate a key barrier identified: providers’ skepticism towards engaging patients and devolving power to them [42, 55]. Therefore, these techniques helped to create a level playing field and support staff in their efforts to be partners.

#### Techniques to enhance representation

With respect to sampling and recruitment, several studies stressed the importance of ensuring diversity and representation consistent with the broader population across different professional backgrounds and skills [43, 45, 54]. These studies endorsed recruiting patients through providers, [42] existing patients [56], and those with broader networks or previous working relationships with staff [45, 54, 57, 58]. These techniques proved useful in engaging patients in the context of HIV/AIDS prevention, interventions to reduce repetition of self-harm and substance use, and identifying barriers to mental health services [42, 56–58]. One caveat with this approach is that it needs to be weighed against the potential for introducing biases or including self-selected participants. Offering stipends, financial compensation (e.g., child care, transportation), or other incentives encouraged participation [42, 45, 50, 54, 55, 58, 59]. One study in the HIV setting used creative techniques to incentivize participation beyond monetary incentives, such as counseling, access to medical care, and granting diplomas [58].

#### Techniques to enhance involvement

Several authors also endorsed flexible approaches for involving patients [45, 49, 53, 54]. For example, Gibson et al. [60] used peer reporter interviews (where patient pairs interviewed each other), headline generation (where phrases were created to capture important issues), group discussion (using a Who, Why, When, What, How structure), a written exercise, and questionnaires for non-attendees to find out what youth would like from their follow-up pediatric oncology services. Other techniques identified in studies were the inclusion of higher proportions of patients compared to providers or staff to give patients a stronger voice in the discussion and process [61] and building in debriefing to provide feedback on how suggestions were acted upon to increase the accuracy of the findings and offer an opportunity for additional input. These techniques proved useful in engaging patients to prioritize stroke service issues and document the process of change of a mental health organization [62, 63]. Others built in regular updates to patient support group to elicit more views, thereby broadening the reach and involvement of patients and providing opportunities to raise and discuss issues of concern in informal settings [48, 54]. One creative technique was a buddy system for users/families to ensure their participation at meetings and throughout implementation/evaluation of a quality improvement project in mental health services [44].

#### Techniques to create a receptive context

Several studies across general medicine, diabetes, mental health, and emergency services highlighted the importance of creating a receptive context by giving each of the stakeholder groups equal say, using techniques such as deliberation and democratic dialog, [39, 51] values and beliefs exercise [48], and narratives to facilitate shared understandings, generate consensus, or find common ground [54, 64]. These techniques created a level playing field and supported staff in their efforts to be partners. Other studies focused on the empowerment and autonomy of users through “active citizenship” in an “egalitarian spirit” [50, 65] which was found to foster a culture of respect [54]. Ensuring that users had an equal voice throughout all aspects of building the intervention was found to help equalize the power differential that often arises in professionally delivered services [65]. Finally, location influenced participation—some studies held consultation outside of the hospital setting such as a disco to appeal to youth [66]. Others conducted meetings in participants’ homes [58] and childcare community sites [67].

External facilitation [39, 63] catalyzed receptive contexts that encouraged user involvement by creating a positive working environment with mutual respect and equal partnership [53]. Finally, attention was also paid to the physical environment (e.g., cleanliness, chair arrangement [53]) and use of physical props, and visual mapping, which supported participants’ discussion and interactions as well as demonstrated to service users the importance of their contribution [68].

#### Leadership actions

A key facilitator of successful engagement was actions and involvement by organizational leaders. This occurred in a variety of ways including top-down approaches and at community levels where local champions led initiatives or were actively engaged to ensure their success. Top-down approaches included institutional- or executive-level commitment and sponsorship, which was readily apparent across mental health, HIV, and pediatric care settings [41, 44–46, 50, 63]. Having managers and executives recognize and advocate for the importance of patient involvement fostered a sense of empowerment and commitment among patients and ensured organizational sustainability of the engagement. This was a goal of two mental health studies, where the senior level of a local authority took a “top-down” approach to promote user involvement, which resulted in a reported culture change throughout the authority [40, 63]. This was highlighted in one study’s “ideological and policy commitment to meaningful involvement of people affected with HIV” as demonstrated by ongoing contact with management and executives and a head clinician open to changes that would disturb traditional relationships and power disparities between service users and providers [50]. Leadership action was also shown to help align the engagement findings or recommendations and ensure that they are advanced within the organization’s relevant strategic plans and policies in primary care [69]. Timing is also an important factor—ensuring that the engagement occurs prior to decision-making, rather than providing input on proposals to which services are already committed was stressed in a number of studies [45]. Otherwise, the engagement could run the risk of being perceived as tokenistic by the users.

### Outcomes of engaging patients to improve quality of care

#### Discrete outcomes of improved quality of care

Most studies noted more than one type of outcome on the quality of care, including enhanced care or service delivery (*n* = 35), development of specific policy or planning documents (*n* = 15), and enhanced governance and education or tool development (*n* = 5 and 11, respectively). Examples of educational materials, tools, policy, and planning documents included evaluation tools [49], electronic personal health records for mental health users [70], a new tool for discharge [71], creation of models of care [72], and organization priorities and processes [49]. Examples of care process, service delivery, and governance included the creation of a prevention of delirium program [73], family integrated program in NICU [45], and care pathway for cellulitis that reduced admissions to hospital [48]. Other engagements in this category led to complete organizational redesign of an outpatient HIV clinic in Southern Norway [50], reconceptualized service for outpatients [68], and revisions to the delineation of roles and responsibilities between an Aboriginal community-controlled health service and local Australian health service [74].

We conceptualized the development of educational materials, tools, policy, and planning documents as “discrete products,” whereas enhanced care process, service delivery, and governance constituted “care process or structural outcomes.” Interestingly, discrete products were more likely to derive from studies using lower levels of engagement (i.e., mostly consultative with elements of co-design), while care process or structural outcomes were more likely to result from higher levels of engagement (i.e., co-design) (Table 3).

#### Impact of engaging patients on the institution

Engaging patients can also change the culture of staff and care settings. The experiences reported in these articles included shifts in organizational culture promoting further patient participation in service design and delivery, [40, 63, 75] achieving collaboration and mutual learning, [42, 47, 76, 77] and sharing or neutralizing power among patients and providers or staff, [52] as well as developing new competencies and negotiating for service changes [39, 59] (Table 4). Interestingly, these outcomes tended to arise in mental health settings and from co-design engagements (Table 5). Further analysis of the methods used in these studies revealed key enabling factors including creating deliberative spaces to share experiences, including external facilitation; broadening power and control to include users, values, and beliefs exercises; conducting user/staff/provider training; and implementing a top-down approach from the local authority (Table 5).

**Table 5:** Examples of studies reporting the impact of engaging patients in institutions

Reference	Level of engagement	Service type	Patient engagement outcome	Method/facilitator
Acri et al. 2014 [65]	Consultative to co-design	Mental health	Shared/neutralized power	Equal voice of users and organization
Barnes 2000 [75]	Co-design	Mental health	Culture change	Educational program
Barnes and Wistow 1994 [40]	Consultative to co-design	Mental health	Culture change	Top-down approach from the local authority
Buck 2004 [76]	Consultative to co-design	General health	Collaboration and mutual learning	Citizen advisory board
Elwell 2014 [48]	Consultative to co-design	Acute care	Organizational impetus to change	User group meetings
Frazier 2007 [42]	Co-design	Mental health	Collaboration between community and clinicians	Service model development
Godfrey et al. 2013 [73]	Consultative to co-design	Acute care	Culture change	Program development
Hopkins and Neimec 2006 [52]	Co-design	Home tx services	Shared/neutralized power	Users conducted research/interviews
Iedema 2010 [39]	Co-design	Emergency services	Development of new competencies	Created deliberative space to share experiences
Jones 2008 [62]	Consultative to co-design	Stroke services	Meaningful user participation	External facilitation
Lord 1994 [63]	Co-design	Mental health	Culture change	Broadening power and control
Macdonnell et al. 2013 [45]	Co-design	Pediatrics	Enhanced relationship between users and providers	Program development
Mendenhall 2010 [77]	Consultative to co-design	Diabetes	Collaboration between community and providers	Talking circles, storytelling, giving users active role
Pilgrim and Waldron 1998 [59]	Co-design	Mental health	Direct negotiations for change	Empowering users and external facilitation
Reeve et al. 2015 [74]	Co-design	Primary care	Enhanced relationships between community and providers	Extensive community consultation
Swarbrick 2006 [55]	Co-design	Mental health	Collaborative partnership	User training
Thomson et al. 2015 [68]	Co-design	Multiple sclerosis	Mutual understanding	Program development
Todd 2000 [47]	Co-design	Intellectual disability	Shift in thinking, collaboration, and participation	Higher proportion of users to providers, training, and clarity of roles
Tollyfield 2014 [53]	Co-design	Acute care	Reconnection to core values of caring and compassion	Ongoing co-design meetings
Tooke 2013 [49]	Consultative to co-design	Dementia	Enhanced communication between users and providers	Service user panels
Walsh and Hostick 2005 [99]	Consultative to co-design	Mental health	User ownership	External facilitation
Xie 2015 [54]	Co-design	Acute care	Commitment and mutual understanding	Familiar, experienced user representatives, establishing common ground and updating users on progress

### Patients’ experiences of being engaged to improve quality of care

Twenty-three of the 48 studies provided information on the patients’ experiences of their engagement, though only 12 studies formally evaluated patients’ experiences in the process of being engaged to improve quality of care. Of those that evaluated experiences, ten studies reported positive views, while in two studies, patients reported negative experiences and two studies reported both positive and negative experiences (Additional file 3: Table S1). Of the positive experiences, patients and carers expressed satisfaction with the engagement processes [43, 78] were interested in continuing their involvement in the longer term, [75] felt the experience to be educational, [52] and felt that participation highlighted issues that would have otherwise been ignored [39, 64, 75]. Positive experiences were linked to feeling empowered and independent as a result of skills development and positive recognition [58, 59, 63]. Some patients reported increased self-esteem from contributing [41, 58, 66] and improved self-efficacy and self-sufficiency [76] and that the experience encouraged peer educators to pursue formal training [55]. In another study, staff reported learning about user participation [46].

Patient feedback in other engagement studies was not as positive. Some studies found that patients were satisfied but felt the engagement demanded considerable energy and time [66]. Others felt that their involvement was tokenistic because decisions had been made in advance or was used to justify decisions that had already been made [47, 61]. Some participants felt that their requests were denied or that managerial support was lacking [47], while others were dissatisfied with their lack of involvement in analyzing the findings and creating the final report [46].

### Quality appraisal

The average quality of the studies was “fair,” based on a quality appraisal tool that systematically reviews disparate forms of evidence and methodologies on a scale from “very poor,” “poor,” “fair,” and “good” [38] (Additional file 4: Table S2). We also assessed the possible impact of study quality on the review’s findings (akin to a “sensitivity analysis” conducted for meta-analyses). There were only 6 (of 48) “poor” quality studies. Removing the six poor quality studies reduced the number/range of examples provided for our findings on the strategies/contextual factors that contributed to optimal patient engagement (research question 1), their outcomes on services (research question 2), and patients’ experience of being engaged (research question 3), but deletion of these studies from the analysis did not alter the substance of the findings.

## Discussion

This study provides a comprehensive review of the strategies used to engage patients in service planning, design, and evaluation. It also identifies the outcomes and contextual factors shaping optimal patient engagement to improve quality of care. Strategies and contextual factors that enabled patient engagement included techniques to enhance design, recruitment, involvement, and leadership action, and those aimed at creating a receptive context. Reported outcomes ranged from developing education or tools for patients and providers and informing policy or planning documents (discrete products) to enhanced care, service delivery, and governance (care process or structural outcomes). Interestingly, the level of engagement appears to influence the outcomes of service redesign: discrete products largely derived from low-level (consultative) engagement, whereas care process or structural outcomes mainly derived from high-level (co-design) engagement. Surprisingly, only a minority of studies (*n* = 12; 25%) formally evaluated patients’ experiences of the engagement activities. While most experiences were positive, some patients sought greater involvement and felt that their involvement was important but tokenistic, especially when requests were denied or when the engagement was used to justify decisions that had already been made. However, it remains unclear how these initiatives affect patients and whether these improvements translate into improved quality of care at a system level.

There were several limitations to this review. Despite the large number of initial search results, there was only a small number of studies focused on involving patients in co-designing health service improvement. Therefore, despite our best attempts, the specificity of our search criteria was modest, a problem familiar to systematic reviews in health services research, which typically crosses many disciplinary boundaries [38]. Future searches would benefit from improved keywords or MeSH terms on the topic of patient engagement. In addition, studies characterized health service users and their involvement differently, ranging from user-centeredness, patient-centered care, and user involvement to patient involvement or participation. Indeed, “user” was a common term used in the UK, whereas other terms such as “patient” and “caregiver” are commonly used in the USA and Canada. These different conceptualizations might signify important distinctions, and the use of different terms, and the publication of these papers across many different journals, raises challenges in identifying and analyzing this literature. We addressed this limitation by using multiple terms and search strategies across multiple disciplinary databases that incorporated terms used in similar reviews. We deliberately sought out the terminology used in key articles to expand our search though may not have captured the entire breadth of terms, such as “consumer,” a popular term used in Australian health services research. We echo previous work that identified this “conceptual muddle” as “one of the greatest barriers to truly integrating patient involvement into health services, policy, and research” [79].

There was also significant variation in sample sizes and populations included in these engagement studies. Samples sizes ranged from 3 to 372 participants and included a variety of patients, families, caregivers, service users, health care providers, staff, board members, health care managers, administrators, and decision-makers. Many studies did not provide details on their sample. These variations illuminate the absence of a standard approach for designing and reporting engagement initiatives. This variation may also reflect the variety of journals in which this research is reported. Additional limitations include the variety of methods used and the limited evaluation of the engagement methods themselves. Where there was no explicit evaluation of engagement, other information including authors’ discussion of strengths and limitations was used to assess the effectiveness of engagement. However, this does not specifically comprise evaluation of the engagement process or its outcomes on care. Development of evaluative metrics and frameworks for the procedural and substantive outcomes of engagements appears warranted. A final important limitation is that our search ended in 2016, and therefore, these insights may differ in the future given the rapidly growing field of patient engagement. This is a limitation familiar to systematic reviews but a future review may be warranted.

Despite these limitations, our study revealed key insights into the factors that influence the ability of health care organizations and decision-makers to create opportunities for engagement that are not provided in individual studies, which cross disciplines and geographical boundaries. We found that successful patient engagement resulted in culture change within the organization, meaningful collaboration and mutual learning, and shared or neutralized power, which tended to arise in settings where co-design is used. Optimal engagement often includes some of the following strategies: use of deliberative spaces to share experiences, external facilitation, broadening power and control to include users in all aspects of the process, flexible approaches for involving users, user training, clarity of roles and objectives, providing feedback, leadership by local champions and securing institutional and/or executive level commitment, and sponsorship from local authority by way of dedicated resources and on-going contact with management and executives. Leadership is key, but there may be a potential temporal trend in leadership actions; top-down approaches to patient engagement tended to be reported in earlier studies [40, 63] whereas more clinician or community-driven initiatives emerged from more recent studies [42, 77]. Another important factor is the timing of engagement. If the engagement occurred after a decision had been made, the success (or even function) of the engagement became highly questionable from the patient’s perspective. Taken together, this analysis suggests that co-design methods supported by executive sponsorship or driven by local champions that use externally facilitated, deliberative, experience-based discourse with trained users can promote successful patient engagement and outcomes.

Mental health settings emerged as a frequent venue for patient engagement in our review. The earliest reports in our review [61, 63, 80] are in this setting, suggesting that the therapeutic approaches, the nature of the population, or the orientation of mental health services might encourage greater patient participation in this area. Indeed, enabling service user involvement in care planning is a key principle of contemporary mental health guidance in the UK [81] and a potentially effective method of improving the culture and responsiveness of mental health services in light of a service history founded on aspects of containment and compulsion, and the stigmatization of those using mental health services [82]. Many of the co-design engagement activities that led to staff and organizational changes such as improved collaboration and mutual learning [42, 47, 76, 77], sharing or neutralizing power among patients and providers or staff [52], developing new competencies, and negotiating for service changes [39, 59] also occurred in mental health. While patient engagement is now occurring in many settings, the experiences in mental health settings serve as important examples of effective patient engagement.

Ultimately, the effectiveness of any patient engagement should be judged by its impact on patient care. There is a growing body of literature that indicates that engaging patients can lead to improved effectiveness, efficiency, quality of care [28–31], health outcomes, and cost-effective health service utilization [27, 83, 84]. The outcomes reported in our review spanned beyond improved care to include enhanced governance and informed policies and organizational planning, which illustrates the breadth of quality of care initiatives that might be sought through patient engagement. However, drawing causal associations between engaging patients in health services improvement and health outcomes is difficult. Furthermore, it remains unclear whether these improvements translate into sustained or improved quality of care beyond local settings at a system level. Indeed, one study found a lack of evidence that patient involvement leads to the implementation of patient-centered care [85]. Some evaluative tools are emerging [86], yet more studies are needed that assess the conditions on which these tools and strategies can sustain the quality of care systemically.

Our review builds upon previous reviews in this field by providing insight into the associations between quality improvement methods and the varying system-level outcomes they yield. Indeed, our review echoes previous research indicating that patient engagement can lead to a multiplicity of health services outcomes with sufficient role definition, training, and alignment of patient-provider expectations but that the quality of the reporting has been poor and the full impact of patient engagement is not fully understood [87–89]. Previous reviews have been limited to specific countries [87], care settings (e.g., mental health [89]), hospitals [90], or study design (e.g., qualitative studies [88]). In this way, our review provides a comprehensive perspective of optimal strategies used internationally, across care settings and using multiple methodologies to engage patients, caregivers, and relatives in quality of care improvement initiatives. Our review also provides novel insights into how the level of engagement influences the outcomes, namely, discrete products (e.g., development of tools and documents) largely derived from low-level engagement (consultative unidirectional feedback), whereas care process or structural outcomes (e.g., improved governance, care or services) mainly derived from high-level engagement (co-design or partnership strategies). If the benefits of engaging patients in the design or delivery of health care are to be realized at an organization or system level, then effective strategies and the contextual factors enabling their outcomes need to be identified so that learning can be generalized. Importantly, our review provides guidance on the effective strategies and contextual factors that enable patient engagement including techniques to enhance the design, recruitment, involvement, and leadership action, and those aimed to create a receptive context.

Future research would benefit from greater consistency in the conceptual, methodological, and evaluative frameworks employed. Greater emphasis is also needed on a procedural evaluation that assesses group composition, group cohesion or collaboration, equality of the participation, and the level of deliberation/reasoning. Such assessments are being developed in the deliberative democracy field [91] and could be informative in patient engagement initiatives. The limited evaluation of patients’ experiences is particularly ironic given the intent of these services to be patient-centered. Additional evaluative metrics should be developed to examine patients’ experiences. Finally, since it is difficult to draw causal relationships between patient engagement and health outcomes, future research should incorporate longitudinal measures and approaches to explore the impact of patient co-design on quality of care.

Several practice implications also emerge and reflect factors linked to the success of quality improvement initiatives more generally. Senior leadership support is critical to success since it increases the likelihood that the relevant decision-makers will implement the findings, and dedicated resources may encourage staff commitment to these efforts.

## Conclusions

Despite the substantive body of research on strategies to engage patients and their effects on patients and health services, the literature is varied and dispersed. This study provides a comprehensive review of the strategies used to engage patients in service planning and design, identifies the outcomes, and contextual factors shaping optimal patient engagement to improve quality of care. Patient engagement can inform education, tools, planning, and policy (discrete products) as well as enhance service delivery and governance (care process or structural outcomes). The level of engagement appears to influence the outcomes of service redesign; discrete products are largely derived from low-level (consultative to co-design) engagement, whereas care process or structural outcomes mainly derived from high-level (co-design) engagement. Further evidence is needed to understand patients’ experiences of the engagement process and whether these outcomes translate into improved quality of care.

## Additional files

Additional file 1: **Table S3.** PRISMA checklist. (DOC 63 kb)

Additional file 2: **Figure S1.** PRISMA diagram. (DOC 57 kb)

Additional file 3: **Table S1.** Analysis of patient engagement strategies to improve quality of care. Identification of facilitators and barriers to patient engagement and subsequent evaluation of patient experiences. (DOCX 160 kb)

Additional file 4: **Table S2.** Quality appraisal. Quality appraisal of review articles based on Hawker S, Payne S, Kerr C, Hardey M, Powell J. Appraising the evidence: reviewing disparate data systematically. Qual Health Res. 2002;12(9):1284–99. (DOCX 117 kb)
