# Supplementary material for: Engaging patients to improve quality of care: a systematic review
Source: Implement Sci. 2018 Jul 26;13:98. doi: 10.1186/s13012-018-0784-z (PMC6060529; doi:10.1186/s13012-018-0784-z)
Supplement: Supplementary file 2 — Figure S1. PRISMA diagram. (DOC 57 kb) [file 13012_2018_784_MOESM2_ESM.doc]

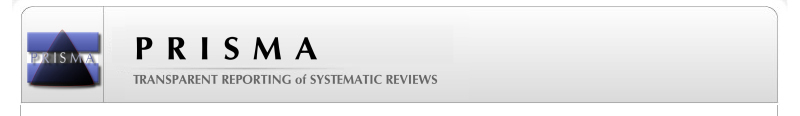
**PRISMA 2009 Flow Diagram**

**Screening**

**Included**

**Eligibility**

**Identification**

Records identified through database searching
(n = 20,947)

Additional records identified through other sources
(n = 10)

Records after duplicates removed
(n = 20,488)

Records screened
(n = 448)

Records excluded
(n = 219)

Full-text articles assessed for eligibility
(n = 228)

Full-text articles excluded, where patients were informed or consulted
(n = 91)

Full-text articles excluded, where reported outomes did not pertain to health care delivery, design or evaluation

(n = 67)

Studies included in qualitative synthesis
(n = 48 )

Studies included in mixed-method synthesis (frequency & thematic analysis)
(n = 48 )
