# Supplementary material for: Engaging patients to improve quality of care: a systematic review
Source: Implement Sci. 2018 Jul 26;13:98. doi: 10.1186/s13012-018-0784-z (PMC6060529; doi:10.1186/s13012-018-0784-z)
Supplement: Supplementary file 3 — Table S1. Analysis of patient engagement strategies to improve quality of care. Identification of facilitators and barriers to patient engagement and subsequent evaluation of patient experiences. (DOCX 160 kb) [file 13012_2018_784_MOESM3_ESM.docx]

***Additional Table 1: Analysis of patient engagement strategies to improve quality care***

| **Study** | **Facilitators identified** | **Barriers Identified** | **Evaluation of Patient Experiences** |
| --- | --- | --- | --- |
| Acri et al.  2014 (65) | Use of community collaborative model that enabled an equalization of power between professionals and peers | Not discussed | Evaluated perceptions of peer support workers as they delivered intervention |
| Barnes et al., 2000 (75) | Meetings with the service users in advance to help them understand how to contribute meaningfully to the process | Users being unable to attend on the day of the commissioning process | *Not formally evaluated*. One user expressed interest in continuing to be involved in the steering group and interview committee |
| Barnes & Wistow 1994 (40) | Clarity about the purposes of involvement and who should be involved  Established mechanisms to act on issues raised and to continue involvement  Strong charismatic leader needed to promote user involvement and culture change  Development of a framework with guiding principles for community engagement Establishment of cross-departmental task groups  Development of city-wide CEO group across departments to discuss overarching community care developments  Buy in across local authorities/depts  On-going resources for user groups for sustainability | Representativeness of users involved – difficulty getting certain groups to participate  Substantial time required  Costs  Concern of being labelled a ‘troublemaker’  Frustration at lack of response or lack of any intention of action  Behaviour change in front-line providers needed to enable user participation | Not discussed |
| Berg et al.  2015 (50) | Establishment of a "user" board  Users defined targets for services  Deliberate recruitment to user board to ensure breadth of representation  Members of user board paid  Regular frequency of meetings  Ongoing contact with management and executives  Encouragement from head clinician for user board to suggest changes challenging traditional relationships between providers and users | Not discussed | *Not formally evaluated.* Informal evaluation of user board members experiences such as through reports to executives |
| Blickem et al. 2013 (91) | Working with local community groups  Workshops providing time to discuss issues  Past experiences | Not discussed | Not discussed |
| Bone et al  2013 (92) | Use of community-based participatory research (CPBR) principles  Diverse representation on community academic advisory committee | Retention of community academic advisory committee members over four-year period | Not discussed |
| Brooks 2008 (64) | Experiential knowledge is a motivator for participation and sustained commitment  Use of narratives facilitated shared understandings, consensus and agenda prioritization. | Professionals’ discounted the value of patients’ narratives/ experiential knowledge | Patients felt that their experiential knowledge was valuable and brought out ideas that professionals would not otherwise have been aware |
| Buck, 2004 (76) | Breakfast provided at each meeting  Meeting reminders | Transportation fees to attend meeting  Job inflexibility of participants  Distance to attend meetings  Child-care responsibilities | *Not formally evaluated.* Reported that homeless participants improved their self-efficacy and self-sufficiency |
| Carlson & Rosenqvist 1990 (51) | Conceptual modelling an effective forum for democratic dialogue and reaching consensus  Selection of participants from different professional categories and consumer input are pre-requisites | Providers outnumbered patients  Inclusion of providers who had cared for patients in the past. | Not discussed |
| Cawston, 2007 (69) | Participation of patients/residents in the research design process  Recruitment beginning within close networks then expanded to the larger community  On-going dissemination of results to residents  Alignment of recommendations with national priorities | Passive dissemination of findings to decision makers limited influence  Limited resources  Undeveloped connections between local units and wider services  On-going organizational reorganization | Not discussed |
| Coad, 2008 (41) | Clear understanding of roles and expectations Flexible approaches to participation  Users set agenda for meetings  Leadership involvement as bridge to organization  Skills training  Built in reward mechanisms such as feedback and evaluation | Participants feeling they were not being taken seriously  Onerous responsibilities  Lack of feedback on how suggestions were acted upon | Youth users felt that their involvement increased their self-confidence and self-esteem and provided valuable experience for their college applications |
| Coker et al 2014 (72) | Not discussed | Not discussed | Not discussed |
| Elwell  2014 (48) | Development of a shared mission and purpose statement through values and beliefs exercise  Enabled opportunity to raise and discuss issues of concern  Regular updates provided to patient support group to elicit more views |  | Used framework for reflection in action to evaluate at all stages of project progression.  Positive views of engagement reported by all stakeholders |
| Ennis et al  2014 (70) | Involvement of users and staff members at all stages in development of electronic health record | Not discussed | Not discussed |
| Enriquez et al. 2010 (67) | Use of Participatory Action Research (PAR) methods  Use of a natural helper model  Use of a childcare site where mothers were already accessing services and building upon existing relationships established in the community  Childcare and transportation provided to participants. | Not discussed | *Not formally evaluated.* Authors indicate that participants may have been more receptive to the intervention since community members implemented it. |
| Erwin et al  2016 (71) | Immersion of investigator with users | Not discussed | Not discussed |
| Factor, 2002 (57) | Identified participants through existing associations with an agency | Not discussed | Not discussed |
| Ferreira-Pinto, 1995 (58) | Skills training and non-cash incentives (e.g., counselling, medical care, granting diplomas)  Recruitment through established outreach networks  Meetings in participants’ homes  Provision of child care  Trained facilitators led discussion  Interactive methods to promote discussion, exploration and ideas | None discussed | *Not formally evaluated.* Reports of increased self-efficacy and self-esteem, empowerment and awareness of risks. |
| Fitzgerald, 2011 (78) | Flexibility, inclusivity and familiarity of approach | Not discussed | *Not formally evaluated*. Satisfaction with process expressed through informal via debriefing sessions |
| Frazier, 2007 (42) | Development of clinician-community partnerships  Selection of community partners who were parents with extensive history in community  Positive relations with community residents and school staff  Clear goals for project  Specified roles  Support for transportation  Provision of child care  Provision of food during sessions  Flexible approaches to participation | Clinician scepticism of user involvement  Tensions about users serving as service partners and community members  Retaining active roles for users in service team  Lack of clearly defined user role | Not discussed |
| Gibson, 2005 (60) | Various flexible approaches to participation to engage participants (peer reporter interviews, headline generation, group discussion, written exercise) | Reliance on staff to recruit users  Workshops run on weekends | Not discussed |
| Godfrey et al  2013 (73) | Not discussed | Not discussed | Not discussed |
| Hall, 2011 (93) | Not discussed | Not discussed | Not discussed |
| Higgins et al  2016 (94) | Direct recruitment through clinician  Ad hoc approach to recruitment of service user or family member facilitators | Noted ethical concerns in recruitment of patients with mental health issues and need for ongoing consent | Not discussed |
| Hopkins & Neimiec 2006 (52) | Participation of all past users  Flexibility and confidentiality of user involvement  Delphi technique allowed users to react to ideas without bias  Training to enable greater participation | Users may not have confidence to participate fully | *Not formally evaluated.* From user interviewers indicated that the experience was educational and were interested in the study’s findings. |
| Iedema et al 2010 (39) | Strong facilitation and dedicated resources Maintenance of relationships with participants  Deliberative co- design processes offers meaningful participation | Recruiting patients and maintaining project momentum  Existing grievances from patients/caregivers negatively affecting the co-design group morale  Unequal proportions of patients and providers affect dynamics. | Satisfaction with the co-design process expressed by all participants  Patients felt that they highlighted issues that would have otherwise been ignored, but patients also felt outnumbered by professionals, resulting in perception that engagement benefited professionals more. |
| Jones, 2008 (62) | Action research framework focuses on users’ experiences  External facilitators ensured progress with service development, maintained user participation, enhanced unbiased perspectives  Strategies used to shift ownership away from researchers to providers to facilitate motivation and commitment  Debriefing exercises to ensure accurate representation and additional feedback. | Providers involved of less influential positions and thus uptake of recommendations is less likely Cognitive difficulties of patients required participation of caregivers instead | Not discussed |
| Jones, 2010 (95) | User was trained and participated as moderator and recruiter for online chat sessions | Not discussed | Not discussed |
| Lofters et al  2015 (43) | Community advisory committee members included members of the targeted population who also represented key community organizations of different sizes and expertise to tap into pre-existing resources | Long duration of participation required | Community advisory committee members were interviewed by an independent party; it was found that participants were generally satisfied with the participatory methods. |
| Lord 1994 (63) | Strategic planning committee included consumers  External facilitation  Post-meeting debriefing  Consumers given equal say  Education sessions during planning process Organizational commitment to change and consumer participation  Management recruited patients to participate, including face-to-face requests | Loss of external facilitation led to reduced implementation, collaboration and concern regarding pace of change  Lack of consumer representation on implementation committee  Staff felt divided between providing own views and supporting consumers on committee  Lack of confidence and health conditions prevented full participation | Evaluation results suggested that consumers felt increased ownership over service provision, increased independence and greater integration within the community |
| Macdonnell et al  2013 (45) | Previous working relationships with NICU staff  Involvement in all stages of project  Parents represented different professional backgrounds and skills  Financial compensation  Flexible environment  Training for parents | Not discussed | Feedback on training used to inform revisions for future training session  Fellow members of the team all reported positive experiences with involving the parents |
| MacNeill 2009 (96) | Action research approach made participants feel comfortable with participation  Use of ‘bonding days’ enhanced understanding of roles and reduced social distance | Lack of accessible information, complexity of discussion and role definition left participants lacking confidence, confused about responsibilities and prevented full participation  Difficulty in recruiting parents to participate disillusioned staff in the importance of participation | Not discussed |
| Mendenhall, 2010 (77) | Community members working with clinicians and researchers to build trust within the local community and sensitize them to cultural issues in the community  Family members collecting each other’s data, which increased trust  Analyses presented to community members by citizen action group | Not discussed | Not discussed |
| Murphy et al 2015 (44) | Senior level engagement  Application of quality improvement methodology together  High level stakeholder commitment  Transparency in process  Positive group dynamics  Stakeholder analysis done at outset to determine membership and roles  Buddy system for users/families to ensure their participation at meetings and throughout implementation/evaluation of project  External facilitator/advisor throughout project | Family/carer focus group was not possible due to time and travel constraints | Interviews with working teams and individual interviews, both with positive feedback about experience |
| Owens, 2011 (56) | Openness to change and flexibility  Ability to change directions with user input Lay team member recruited other service users  Hourly payments provided for attendance Latitude and open-mind increases successful development and implementation of service | Inconsistent participation of users due to health issues, which required re-establishing aims and consensus each meeting  Power balances may shift over course of participation, depending on time and skills demanded by tasks  Longer timeframes needed in development phase | Not discussed |
| Pilgrim & Waldron 1998 (59) | Recognition of patient involvement by facilitators and managers fostered empowerment  Honorariums encouraged participation | Some issues generated by the group had variable degrees of external constraint, thus were less actionable  Economic constraints impacted self-esteem when negotiating with managers for funding | *Not formally evaluated*. Authors suggest group felt empowered as a result of skills development and positive recognition |
| Reeve et al 2015 (74) | Shared vision and trusting relationships resulting from the community consultation  Local community leadership | Not discussed | Not discussed |
| Rose, 2003 (97) | Users developed and conducted surveys | User-researchers may be selective in recruiting as first point of contact, biasing the sample  User-researchers were resistant towards administering survey to other users | Not discussed |
| Sawbrick, 2006 (55) | Stipends provided to peer educators | State level policies and procedures  Transportation challenges for peer educators to attend meetings  Training and education of peer educators  Scepticism from staff about having mental health patients as peer educators | *Not formally evaluated.* Anecdotal report that participating has encouraged peer educators to pursue formal training |
| Thomson  2015 (68) | Use of Design Researcher to facilitate future group sessions  Use of physical props and visual mapping supported participants discussion of future interactions  Methods demonstrating the importance of their contribution to service users |  | Qualitative analysis of output of future group sessions |
| Todd 2000 (47) | High level of patient representation versus providers and staff led to greater influence  Providing clarity on roles and responsibilities helped users to understand how they could best contribute | Stakeholders’ perceptions of carers as resistant to change Carers were better at providing pragmatic versus strategic advice | Users felt that their participation was important but tokenistic. Carers felt that their requests were denied. Carers felt that their involvement was used to justify decisions that had already been made by authorities, despite feeling that they knew what was best for service users |
| Tollyfield  2014 (53) | Facilitated by staff with experience in setting and quality improvement methods  Allowed attendance to be flexible for patient/family participants  Preparation and planning for each event and  co-design meeting  Physical environment clean and comfortable, with chairs  arranged for easy line of sight to among all participants | Not discussed | Feedback forms created and accepted verbally  Patients reported participating was a positive experience, as did staff |
| Tooke  2013 (49) | Development of relationship between facilitator and participants  Allowing sufficient time for relationships to develop before process bean  Engaging with service users in a location of their preference  Enabling panels to identify themselves with a name and choose topics to discuss based on submissions from staff or other panellists  Meeting materials adapted for population (e.g. using graphics and large font) | Use of facilitator resulted in some participants giving the answers they felt were expected of them | Service user panels evaluated through participant observation and focus groups with the panels  Staff who visited a panel to discuss a project were also interviewed about experiences  Facilitation of service user panels may result in limitations on extent to which they are user-led  Service user panels rely on staff to act on feedback. |
| Van Staa, 2010 (66) | Held consultation outside of hospital in a setting appealing to, but not easily accessed by, chronically ill youth (discotheque). Recruitment of co-designers by regular care providers as oppose to researchers  Invited participants to bring friends to increase comfort with participation  Provided monetary incentives for co-designers and other incentives for interview participants | Difficult to establish meetings with co-design participants  Limited number of attendees  Time intensive | Though demanding energy, enthusiasm and time, co-researchers felt empowered by their experience. Co-designers reported increased self-esteem and status by earning some money |
| Walsh & Hostick 2005 (98) | Consumer-controlled but professionally facilitated approach  Distant, postal format of consulting providers maintain user confidence in engagement | Financial constraints curtailed support to team and facilitator  Personal conflicts | Not discussed |
| Weinstein 2006 (46) | User-led review process increased sense of ownership  Organizational commitment  Staff representation on steering committee helped with implementing recommendations | Lack of managerial capacity to act on recommendations from users  User-led process made staff feel undermined  Minimal involvement of staff in questionnaire development resulted in a less targeted survey with a lower response rate | Users expressed appreciation of being involved in the process planning but were dissatisfied with their lack of involvement in analysing the findings and creating the final report |
| Wistow & Barnes 1993 (61) | User-led models of engagement; Meeting agendas and structures set by carers allows them to define the issues and increases participation; Providers deliberately kept at arms length to give users a voice and prevent them from overpowering discussion. | Lack of provider involvement could distance management, create difficulty in influencing change. | *Not formally evaluated.*  Anecdotal feedback from users that providers did not learn from their contributions and decisions had been made in advance of user involvement. Enhanced self-esteem of users who participated. |
| Xie  2015 (54) | Multiple meetings  Demonstrated progress between meetings to bring back to team  Ensuring participants were caught-up if a meeting was missed  Representation of all relevant stakeholders  Accommodating scheduling  Incentives for attendance  Establishment of common grounds to overcome differences of opinion  Parent representative possessed relevant inpatient experience in the organization and was familiar with the practices and language of clinical staff  Parent representative had also previously participated in healthcare quality improvement, had experience in advocacy and was known to the other team members | Consistent representation from all stakeholders | Phenomenological approach using semi-structured interviews to understand experience of collaborative redesign process, perspectives on participation of the team members and factors influencing collaboration |
