# Supplementary material for: Engaging patients to improve quality of care: a systematic review
Source: Implement Sci. 2018 Jul 26;13:98. doi: 10.1186/s13012-018-0784-z (PMC6060529; doi:10.1186/s13012-018-0784-z)
Supplement: Supplementary file 4 — Table S2. Quality appraisal. Quality appraisal of review articles based on Hawker S, Payne S, Kerr C, Hardey M, Powell J. Appraising the evidence: reviewing disparate data systematically. Qual Health Res. 2002;12(9):1284–99. (DOCX 117 kb) [file 13012_2018_784_MOESM4_ESM.docx]

***Additional Table 2: Quality appraisal***

| **Study** | **Abstract & title** | **Introduction & aims** | **Method & data** | **Sampling** | **Data analysis** | **Ethics & bias** | **Finding and/or results** | **Transferability and/or generalizability** | **Implications & usefulness** | **Comments** |
| --- | --- | --- | --- | --- | --- | --- | --- | --- | --- | --- |
| Acri et al. 2014 (62) | 4 | 4 | 4 | 3 | 3 | 1 | 4 | 4 | 4 | Sampling: participants involved in intervention design are mentioned (i.e. Family peer advocates and caregivers for delivery of intervention) but there are no details of how they were recruited; Analysis: themes resulting from analysis of interviews outlined but there is no in depth discussion of how they were derived; Ethics: No mention of consent or ethical approval, bias etc.; Findings: Description of the resulting intervention is present; Transferability: the setting and context are described in the introduction as well as overview of the methods |
| Barnes, 2000 (72) | 4 | 4 | 3 | 4 | 3 | 3 | 4 | 4 | 4 | Introduction has background, identifies the gap in care and aims; Details of sample, how and why they were recruited are given; Analysis: Poor/fair, "an analysis of documents concerned with the tendering, both to provide the education programme"; There is a note on ethics and confidentiality on page 11; Findings are in a logical progression, data is presented to support findings, lots of description; Implications missing discussion of future research |
| Barnes & Wistow 1994 (37) | 4 | 3 | 3 | 3 | 3 | 1 | 3 | 4 | 4 | Method is evaluation of CCSAP, outlines areas that were the focus of the evaluation but doesn’t provide explicit details; Introduction: some background, more literature could be provided; Sampling: only mentions that the sample is people with mental health issues/disabilities, types of service users but doesn’t go beyond that; Analysis: briefly describes analytical framework/themes; Transferability: provides good context; Ethics: no mention; Implications: discussion is extensive, whole paper is about implications to this program/service; Findings are accounted for but could be better organized or supported by quotes from interviews/focus groups |
| Berg et al. 2015 (47) | 4 | 4 | 4 | 4 | 3 | 3 | 4 | 3 | 4 | Introduction: aim and gaps in care identified, literature and background are present; Sample: i.e. Of documentation outlining user and provider views, types of and number of each type of documentation are given; Analysis of documents through sorting and systematically reviewing (one sentence only, not very detailed); Results: of analysis of clinic initiative are outlined in detail on pages 4-7; Author recognizes limits to transferability in conclusion; Ethics: subjectivity of the researcher is mentioned |
| Blickem et al. 2013 (82) | 4 | 4 | 4 | 4 | 4 | 4 | 4 | 4 | 4 | Full, structured abstract with clear title; Clear description of method for each stage, data collection and recording; Sample: includes demographics, why and how they were recruited; Analysis: discusses coding and how themes were derived; Ethics: outlines ethics board approval and participant consent, sensitivity of working with people with LTCs; Findings: clear, in logical progression (i.e. for each stage, then for pilot PLANS tool), sufficient data provided, data is also summarized nicely in tables (i.e. box 2); Transferability: NPT specifically applied to PLANS in this article but methodology can definitely be applicable in other contexts; Implications for practice discussed, discusses future potential use of PLANS |
| Bone et al. 2013 (83) | 4 | 4 | 4 | 4 | 4 | 2 | 4 | 4 | 4 | Title is specific and abstract is structured with all necessary information present; Full background with literature review and aims, identifies gaps in literature; Methods are described on pages 3-5; Sampling: details of individuals involved in development are provided, recruitment of CAC members outlined as well as demographics given, drop out rates and reasons given; Analysis: "evaluation of CHWs/navigators"; Ethics: mentions equal treatment of CHWs, cultural sensitivity, no mention of ethical approval or consent; Findings: "barriers identified" and "lessons learned"; Transferability: information is useful for transferring methodology to other patient navigator programs; Discusses implications for practice as well as suggestions for future research |
| Brooks 2008 (61) | 4 | 4 | 4 | 4 | 3 | 3 | 4 | 4 | 4 | Method clearly outlined; Details of sample and recruitment clearly outlined; Analysis briefly described; Ethics: mentions obtainment of written consent |
| Buck, 2004 (73) | 4 | 4 | 4 | 4 | 4 | 4 | 4 | 4 | 4 | Introduction: method is that they formed a board; Details of sample given and how they were recruited and why; Transferability is high because substantial background and context is given |
| Carlson & Rosenqvist 1990 (48) | 4 | 4 | 4 | 4 | 3 | 1 | 4 | 4 | 4 | Analysis is mentioned only; No mention of future research areas; No mention of consent or ethical approval |
| Cawston, 2007 (66) | 4 | 3 | 4 | 4 | 4 | 3 | 3 | 4 | 3 | Introduction: Background could be more detailed, literature could be more recent, aim and research questions are present; Sample was detailed and justified; Analysis: discussion of how themes were derived is present (i.e. integration of personal experiences, comparison on page 3); Speaks to prejudice and labelling, class division between professional staff and participants, no mention of researcher's bias, obtained ethics approval, discussion of relationship between researcher and participants; No quote provided to support second finding (data not completely sufficient to support findings); Study reports limited impact at a local level |
| Coad, 2008 (38) | 4 | 4 | 4 | 4 | 3 | 3 | 4 | 4 | 4 | Methods: clearly outline how they set up the youth council; Analysis briefly described on page 4; Advising on consent and REB approval mentioned as output of council in table 2; Findings are in a logical progression and are also presented in a table, supported with quotes/comments; No suggestions for future research |
| Coker et al. 2014 (69) | 4 | 4 | 4 | 4 | 4 | 1 | 4 | 3 | 4 | Introduction: identifies gap in care and solution, background, literature, study objective; Methods: are summarized and then each step is detailed nicely in subsequent paragraphs; Sampling: Study participants are outlined and described in Table 1 as well as under specific sub heading; Analysis described and break even analysis provided; Ethics: ethical considerations/consent/REB approval not mentioned; Findings: logical progression and are outlined in detail for each phase of the project, also gives table detailing all the models; Transferability: process and model applicable to other clinics interested in redesign, however it is noted in limitations that the WCC models are specific to practices involved in the study and may not be generalizable to others (especially practices that do not serve low income families); Implications for practice discussed |
| Elwell 2014 (45) | 4 | 4 | 4 | 3 | 3 | 2 | 3 | 3 | 3 | Introduction: contains background, literature, gaps in care, objectives/aims; Method: described and also summarized in a flow chart, details of data collection and recording (i.e. On post-its); Sample description could be better, no details of demographics of participants or how they were recruited but they do identify the key stakeholders involved (i.e. nurses, A&E medics); Analysis: mentions constant evaluation of program using framework for reflection in action but does not provide details on how the analysis was done; Ethics: a couple words mentioning consent to use clinical photography but no other considerations; Findings: table of questionnaire results provided but more explanation/ discussion of the findings could be given in the text; Transferability to other contexts may be limited by the fact that this study is related to disease-specific population (cellulitis); Implications for practice could have been discussed further, they calculated monetary savings as a result of the intervention |
| Ennis et al. 2014 (67) | 4 | 3 | 4 | 4 | 3 | 4 | 4 | 4 | 4 | Introduction: Background could be more detailed, but they identify gap in care, there is an aim and literature review; Methods: procedures are outlined for each stage; Sample: Service users are outlined, sample size and how they were recruited, demographics for stage 3 also given; Analysis: briefly mentioned, content analysis for focus groups and Fischer's test/t tests for quantitative data; Ethics: ethical issues, board approval, participant consent discussed, privacy issues discussed; Findings: presented for each stage in logical order, sufficient findings given; Transferability: adequate details of sample and setting given to allow for context, process relevant for designing ePHR for marginalized groups; Implications for practice and need for more research discussed |
| Enriquez et al. 2010 (64) | 4 | 4 | 4 | 4 | 4 | 3 | 4 | 4 | 4 | All other elements are good (abstract and title are sufficient, methods and data clearly explained, sampling is outlined such that it can be repeated so also good transferability, analysis is described, implications are outlined); Ethics: mentioned obtainment of informed consent but nothing else. |
| Erwin et al. 2016 (68) | 4 | 4 | 4 | 4 | 4 | 4 | 4 | 3 | 4 | Introduction: background, literature, gap in care and how the research fills it, aim present; Methods including data collection and recording are outlined for each phase and summarized nicely and visually in figure 1; Analysis described "produced 8 themes…", briefly mention software used for analysis, talk about coding and inter-coder reliability; Discuss ethical conduction of research, board approval, consent; Findings: multiple tables and figures presented to support and discuss findings, presented in logical progression for each phase of research; Transferability: the target population for this intervention is very specific (African/Latino children with asthma etc.) but the methodology is applicable in different contexts; Implications for use of tool in clinic/practice is what the whole paper is describing |
| Factor, 2002 (54) | 4 | 4 | 4 | 4 | 3 | 4 | 4 | 4 | 4 | Methods: included focus group questions; Analysis: there were predetermined themes, descriptive discussion of analysis; Ethics: there was a discussion regarding bias; implications for practice and suggestion for future research given; Findings were well laid out and there was sufficient data to support them in form of table |
| Ferreira-Pinto, 1995 (55) | 4 | 4 | 4 | 4 | 3 | 1 | 4 | 4 | 4 | Very clear aims and a great background/literature review; No mention of ethics board approval or consent |
| Fitzgerald, 2011 (75) | 4 | 4 | 4 | 3 | 2 | 1 | 3 | 4 | 3 | Abstract includes method and type of participants; Introduction: good literature review and background, identifies gaps and how to address them, research objective was to create serious game to address outlined issues/gaps; method: How the data was collected is described, detailed description of how game was created; Sampling: no demographics given but sample size was justified; Analysis: no real discussion of analysis but there is mention of themes and where they got them from; Suggests area of future research |
| Frazier, 2007 (39) | 3 | 3 | 4 | 3 | 2 | 1 | 4 | 4 | 4 | Abstract: what were the actual results of implementing this program?; Introduction provides background and aim but literature review is out-dated and not provided (i.e. it says "extensive literature" but then does not provide any references to back up claim); Steps of developing PALS program are logical and outlined throughout the article; Sample size mentioned but no details of participants given, sample size not justified; Analysis: not explicitly mentioned, on-going as the program was developed (i.e. through on-going discussions, reflection), no indication of how parent opinions were collected; Findings are logically presented as the program is developed, lessons learned are listed clearly; Implications for the classroom/practice outlined |
| Gibson, 2005 (57) | 4 | 4 | 4 | 4 | 4 | 3 | 4 | 4 | 4 | Ethics: did not acknowledge bias |
| Godfrey et al. 2013 (70) | 4 | 4 | 4 | 4 | 4 | 4 | 4 | 3 | 4 | Clear title and structured abstract; Full background with literature review, gap in care identified and study objectives outlined; Methods, including clear description of data collection and recording, are outlined; Sampling: details of sample and hospitals recruited/recruitment procedures outlined; Analysis discussed in depth (page 3-4), grounded theory, coding, etc.; Ethics board approval, informed consent, sensitivity of delirium patients discussed; Findings are discussed in depth and in logical progression with sufficient data presented within the text as well as in tables; Transferability: This intervention is specific to delirium prevention but the methodology is generalizable to other settings |
| Hall, 2011 (84) | 4 | 4 | 4 | 4 | 4 | 4 | 4 | 4 | 4 | Study setting provided for high transferability rating; researcher discussed potential bias in data |
| Higgins et al. 2016 (85) | 4 | 4 | 4 | 4 | 3 | 4 | 4 | 4 | 2 | Introduction: Background literature and objectives clearly outlined and detailed; Clear methods section; details of recruitment, who/how and number of participants given; clear section on data collection and analysis however analysis description was very surface level; There is an entire section on ethical issues; Findings are explained/discussed and supporting data/quotes are presented; Implications for policy/practice are not really discussed, there is no discussion section however findings include suggestions for practice derived from patient experiences with system, indicates that outcomes will be discussed in a second paper |
| Hopkins & Neimiec 2006 (49) | 4 | 3 | 4 | 4 | 4 | 4 | 2 | 3 | 3 | Title is very clear; Introduction: aims are clearly stated, there is some background but literature review isn't very recent, identifies gap in care; Sample: Response rates given (and all other points except no demographics); Thematic analysis is described for both parts of the study; Actual findings from the interviews are not discussed, there is a brief mention of areas where the users offered feedback on parts of the questionnaire (data mentioned but not described) on page 45; No suggestions for future research |
| Iedema et al 2010 (36) | 4 | 4 | 4 | 3 | 3 | 1 | 4 | 4 | 4 | Introduction: extensive background given, aims of paper outlined on page 3; methods are described on page 6; Sample: minimal details given on page 6; Analysis: minimal details; Transferability: describe the process of co-design such that it can be applied to other contexts; Implications: does not discuss future research but provides valuable insight on the co-design process and implications for practice |
| Jones et al 2008 (59) | 4 | 4 | 3 | 3 | 4 | 4 | 4 | 4 | 3 | Data: No interview/focus group questions provided; Sampling: response rates not shown/explained; addresses ethical issues and acknowledges potentially researcher bias; Implications: does not discuss future research possibilities |
| Jones, 2010 (89) | 4 | 4 | 4 | 4 | 2 | 3 | 4 | 3 | 2 | Abstract: title clear, method present, aim; Introduction: aim, full background and lit review as well as clear aim; sample detailed and how they were recruited for each phase outlined; Analysis: There is not really a discussion of analysis, no analysis of themes from interviews; Informed consent mentioned for each phase; Findings: findings are in a logical progression as they are presented after each stage of web site development; Transferability: context is very clearly outlined but not sure about transferability since this is for a very specific population (i.e. deaf community); Contributes something new/novel but missing suggestions for future research and implications for policy/practice beyond that of developing an aid for deaf individuals |
| Lofters et al. 2015 (40) | 4 | 4 | 4 | 4 | 4 | 4 | 3 | 4 | 3 | Methods: outlined for each stage of the KE process in figure 1; Data analysis: "CAG process evaluation," third part conducted analysis via interviews with CAG members; Ethics: board approval and third party consultant to analyse because of potential bias of research team members; Findings: Could have provided more data/quotes to support findings; Implications: the discussion was more about implications for the CAG than large scale implications on practice, although they do talk about it. Did not discuss areas for future research or policy implications. |
| Lord et al 1998 (60) | 4 | 4 | 4 | 3 | 3 | 1 | 4 | 4 | 4 | Introduction: very clear aim, extensive background; methodology outlined under clear heading; Sample: Mentions types of individuals in board/committee members; analysis: "three themes emerged from analysis of case study" page 14; Transferability: setting and context and described in detail; No suggestions for future research |
| Macdonnell et al. 2013 (42) | 4 | 4 | 4 | 4 | 3 | 3 | 4 | 4 | 4 | Surface level description of data analysis; Methods are outlined separately for each 'objective'; Creation of Ethics board applications and consent forms mentioned |
| MacNeill 2009 (87) | 3 | 3 | 4 | 3 | 4 | 3 | 4 | 3 | 3 | Abstract: Definition of “sure start” is unclear, sample size is not mentioned; clear aim, good background, research question not obviously stated; no response rates given |
| Mendenhall et al., 2010 (74) | 4 | 4 | 4 | 3 | 4 | 3 | 4 | 3 | 4 | Introduction: there is an aim statement and extensive background, the research question is clearly stated in on page 365; no response rates given |
| Murphy et al. 2014 (41) | 4 | 4 | 4 | 3 | 3 | 4 | 4 | 4 | 4 | Introduction: not all under the subheading "introduction," but gives background, gaps in care, literature and aims; Sampling: sample size mentioned but no details given (i.e. Demographics not present), little to no details of how participants were recruited; Analysis is mentioned but it is surface level/descriptive; Paragraph on ethics; Transferability: setting and context described |
| Owens, 2011 (53) | 4 | 4 | 4 | 4 | 3 | 2 | 4 | 4 | 4 | Introduction: has literature review, background, gap in care and potenital solution, aim; Methods: clear description of method including data collection and recording; Sample: includes how participants were recruited and why, as well as table of demographics; Analysis: descriptive discussion of thematic analysis, doesn't dive deep, mentioned very briefly in methods section, brief mention of "ethics of raising expectations page 5; Findings presented logically and emergent themes were numbered and discussed for each 'section'; Transferability: enough context and background provided to replicate; Implications for practice clear, developed a model, future research will involve testing the intervention |
| Pilgrim & Waldron 1998 (56) | 3 | 3 | 4 | 4 | 4 | 3 | 4 | 4 | 3 | Abstract: Title could be better, does not outline results (achievements and limitations of the exercise); Introduction: Background is there but it is not extensive; Method is simple and explained clearly; Sample: sample numbers given, recruitment outlined, drop out rate given; Analysis: the group's rate of achievements and difficulties was analysed in context based on prevailing political and social factors (interpretive framework used); Ethics: ethical issues are briefly discussed during analysis but there is no mention of obtainment of consent from participants; Findings: the findings (success and failures of the group) were presented for three items, list of priorities generated by group presented in table; Transferability: the context of this study is described in depth; Implications for policy and practice are mentioned within the discussion of context but could be outlined clearer along with suggestion for future research |
| Reeve et al. 2015 (71) | 4 | 3 | 4 | 3 | 3 | 3 | 4 | 4 | 4 | Introduction: Background is brief, could be more literature review/more recent literature review; Sampling is mentioned (i.e. Number of participants) but few details are given (i.e. On recruitment, demographics); Brief mention of analysis; Ethics: mention of ethics approval; Present data in many tables to support findings, tables are mentioned and explained in text; Context and setting are described in depth for this specific population to allow for comparison to other, similar contexts |
| Rose, 2003 (88) | 4 | 4 | 4 | 4 | 4 | 3 | 4 | 4 | 4 | Introduction: background, gaps in care and solution, literature, aims all present; Methods: outlined and includes survey instruments; Sample: many details given; Analysis: clear, includes significance calculations; Ethics: lip service was given to sample bias and ethics of users interviewing other users, no mention of consent; Findings are sufficient, presented in tables mentioned in text; Transferability: sample is described in depth enough to allow for replication in a similar setting; Implications for policy/practice, limitations of study, future research included |
| Swarbrick et al. 2006 (52) | 3 | 2 | 4 | 3 | 2 | 1 | 3 | 3 | 4 | Title isn't as clear as it could be, what were some of the outcomes of this program?; Introduction: aim is clear, gap is clear, there is background but no literature review (author cites herself); Methods are described and includes table with recovery network discussion topics; Sample: persons in recovery presenting to patients but no details are given, discusses challenges of recruitment; Analysis of the program was in the form of challenges and accomplishments faced by the program but there is no outline of how the data was recorded or what method of analysis was used; Findings: Outcomes are discussed under challenges and accomplishments sections; Implications of program are discussed, suggests further evaluation of program in conclusion |
| Thomson et al. 2015 (65) | 4 | 4 | 4 | 4 | 3 | 4 | 4 | 4 | 4 | Extremely detailed description of methods; Recruitment details outlined under subheading; Brief, descriptive discussion of analysis at the end of 'future group procedure'; Ethics: mentions ethical approval, participant consent, and ethical considerations to be taken into account for the study; Details of setting and recruitment procedures are described and allow for comparison to similar contexts |
| Todd et al 2000 (44) | 3 | 3 | 4 | 3 | 3 | 2 | 4 | 4 | 4 | Abstract: the aim is not clear, I believe it is to reflect on the strengths and weaknesses of policy implementation but it is written like it is part of the methods/interview questions; Introduction: background and aims are provided but literature review is lacking (not many references at all and some are out-dated); Methods: clear descriptions of data collection and recording; Sampling: Only outlines that participants were key stakeholders and from what 'agencies,' no other details of sample given, doesn't outline how they were recruited; Analysis: brief, descriptive sentence of analysis; Ethics: Does not discuss consent, brief mention of welfare of individuals with mental disabilities, equal engagement of service users in policy development; Findings: in logical progression with plenty of quotes to support findings in text; Transferability: specific to Welsh community but AWS is an example of a movement that is similar in multiple developing countries and the study can therefore be applied more widely; Discusses in depth implications for policy and service development |
| Tollyfeld 2014 (50) | 3 | 4 | 4 | 4 | 2 | 1 | 3 | 4 | 3 | Abstract: title is sort of vague, abstract contains most information but there is no background as to why the study is being conducted; Methods described well and summarized in table 2; Sampling described well under sub heading "engaging of staff and patients"; Analysis: It is not clear if interviews were transcribed, in table 2 it says "thematically analysed responses" but nothing is mentioned in the body of the manuscript, there is mention of meetings being 'reflected upon' to generate changes for subsequent meetings; Ethics: no mention of ethics approval, consent, bias; Findings/outcomes are described (however briefly) but little evidence is provided (i.e. quotes) to support; Implications: discussion on implications for overall practice is brief under "conclusions and recommendations" |
| Tooke 2013 (46) | 4 | 4 | 4 | 4 | 4 | 4 | 4 | 3 | 4 | Sampling: details of sample not collected but rationalized by note 2; Models of consent and ethics discussed; Transferability: Specific to people with dementia and setting not really described (only says rural/urban area); |
| Van Staa et al. 2010 (63) | 4 | 4 | 4 | 4 | 3 | 4 | 4 | 4 | 4 | Structured abstract with full information; Introduction: Has full background, literature review, identifies gap, aim; Methods are extensive and detailed; Sampling: Participant details give, recruitment procedures described; Analysis: Brief descriptive, could have had more details of how outcomes (i.e. common themes derived from transcripts, research quality); There is a paragraph on Ethics for the study, obtainment of consent is mentioned, bias mentioned in discussion, sensitivity mentioned in results section; Findings: presented in logical order and summarized nicely in a table, easy to understand; Transferability: sampling was detailed and context was described; Implications for practice and PR discussed |
| Walsh & Hostick 2005 (89) | 3 | 4 | 4 | 4 | 3 | 3 | 4 | 4 | 4 | Abstract: The title is not clear, what is OR? What is NHS? Do not outline acronym in abstract either; Intro: COR background provided, need for more public participation outlined, aim /objective of paper outlined (develop consumer led research groups: two in particular are outlined); Method: Methods are outlined throughout the entire paper and for the two 'research groups'; Sampling: members of each research group outlined, target participants for each research group outlined and how they were recruited (i.e. Trailblazer contacted 50 GPs and 10 responded); Analysis: inquiry tool (table 2) has an evaluation step and analysis within the Trailblazing process (Figure 1 and 2) involves discussions and interpretation of responses... Was not very clear though and I was originally going to rate as 2 but upon detailed reading of the article decided that analysis was accounted for; Ethics: brief mention of ethics, consent; Transferability: These are frameworks which can be applied in multiple settings; Implications for policy and practice discussed |
| Weinstein 2006 (43) | 4 | 4 | 4 | 4 | 3 | 4 | 4 | 4 | 4 | Structured abstract with full information; Introduction: full background, identifies gap, literature review, study/project objectives; Methodology outlined clearly; Sample: The study was a retrospective analysis of documentation from 1998 and 2000, types of documents/QA tools are listed; Analysis: i.e. of retrospective documents is mentioned, but how they were actually analysed is not discussed; Ethics: Author acknowledges own bias and mentions it was determined that no ethical procedures were pursued at a formal level; Findings: Outcomes of each retrospective review are presented clearly and in logical order, summarized in table; Transferability: Setting is described and author mentions relevance for use in similar settings; Implications for practice, governance and ideas for future research discussed |
| Wistow & Barnes 1993 (58) | 4 | 4 | 3 | 3 | 4 | 2 | 4 | 3 | 4 | This was a really tough one to appraise with this framework; Introduction: contains background and aims of paper, furthered background and literature review is provided in subsequent paragraphs discussing the '3 issues'; There is a description of methods of the Birmingham action project on page 10 but not many details are provided about surveys/consultations; Sampling: long term mental health service users, informal carers, individuals with learning disabilities, no other details given; Analysis: i.e. of Birmingham action project using the author's framework is done throughout the latter end of the paper/essay, identify key terms of voice, consultation, influence; Ethics: no mention of consent, ethical approval in reference to the action project but does discuss sensitivity of services to users' needs; findings: Outcomes of involvement are listed and discussed in depth (page 14 onward); Transferability: debate between 3 and 4... chose 3 because of sample rating as well as specificity of analysis to one specific project; Implications for practice and user involvement are discussed in depth, development of more sensitive services, application to other contexts |
| Xie et al. 2015 (51) | 4 | 4 | 4 | 4 | 4 | 2 | 4 | 4 | 4 | Ethics: brief mention of potential bias in data in discussion section, no mention of consent or ethics approval |
| Total average quality | 4 | 4 | 4 | 4 | 3 | 3 | 4 | 4 | 4 |  |

Legend:

4=good

3=fair

2=poor

1=very poor

(Based on Hawker S, Payne S, Kerr C, Hardey M, Powell J. Appraising the evidence: reviewing disparate data systematically. Qual Health Res. 2002;12(9):1284-99.)
